# Transformation of mouse T cells requires MYC and AKT activity in conjunction with inhibition of intrinsic apoptosis

**DOI:** 10.18632/oncotarget.25113

**Published:** 2018-04-20

**Authors:** Kari Högstrand, Stephanie Darmanin, TachaZi Plym Forshell, Alf Grandien

**Affiliations:** ^1^ Center for Hematology and Regenerative Medicine, Department of Medicine, Karolinska Institutet, Karolinska University Hospital-Huddinge, 141 57 Stockholm, Sweden

**Keywords:** peripheral T-cell lymphoma, transformation, apoptosis, quiescence, AKT

## Abstract

Peripheral T-cell lymphoma is an aggressive non-Hodgkin's lymphoma characterized by excessive proliferation of transformed mature T cells. The number and nature of genetic aberrations required and sufficient for transformation of normal T cells into lymphomas is unknown. Here, using a combinatorial *in vitro*-approach, we demonstrate that overexpression of MYC together with activated AKT in conditions of inhibition of intrinsic apoptosis rapidly resulted in transformation of mature mouse T cells with a frequency approaching 100%. Injection of transformed cells into mice resulted in rapid development of aggressive T cell lymphoma, characterized by spread to several organs, destruction of tissue architecture and rapid death of the animals. TcR-sequencing revealed a polyclonal repertoire of tumor cells indicating that co-expression of MYC, activated AKT and BCLXL is sufficient for tumor transformation and do not require acquisition of additional genetic events. When analyzing cells with inducible expression we found that proliferation of transformed T cells required sustained expression of both MYC and AKT. AKT exerted a dual function as it inhibited induction of, and promoted exit from, cellular quiescence and contributed to inhibion of apoptosis. Downregulation of AKT and/or MYC together with BCLXL resulted in rapid and complete elimination of cells through induction of apoptotic cell death.

## INTRODUCTION

Peripheral T-cell lymphomas (PTCLs) or mature T-cell lymphomas are a group of usually aggressive non-Hodgkin lymphomas (NHLs) accounting for around 10-15 % of NHL cases [1–3]. The disease is associated with a poor outcome and there is need for improved treatment regimens. Development of new treatments should be facilitated by an improved knowledge about the molecular mechanisms underlying the disease. PTCLs are highly heterogeneous, consisting of several subgroups. The three most common subgroups, PTCL not otherwise specified (PTCL-NOS), angioimmunoblastic T-cell lymphoma (AITCL) and anaplastic large cell lymphoma (ALCL) account for around 60% of the cases. ALCL can further be divided into two subsets based on presence or absence of recurrent translocations involving the anaplastic lymphoma kinase (ALK) at 2p23 [4]. A subset of PTCL-NOS carry a recurrent translocation t(5:9)(q33;q22) creating a fusion between the *ITK* and *SYK* genes resulting in the expression of the ITK-SYK fusion protein [5]. Recently, several recurrent mutations have been identified in AITCL as well as in a subset of PTCL-NOS with an AITCL-associated gene expression profile. These include mutations in the GTPase RHOA [6, 7] as well as in the epigenetic regulators TET2 [8, 9], DNMT3A [9], and IDH2 [10]. Whereas mutations in RHOA appear specific for AITCL, mutations in TET2, DNMT3A and IDH2 are also common in myeloid malignancies [11, 12]. Mutations in a number of genes including PLCG1, CD28 [13], FYN [7], CTNNB1 (beta-catenin) [13], STAT3, JAK1 and TP53 [14] as well as translocations involving IRF4 [15] have also been identified in PTCL.

Furthermore, deregulated expression of a large number of genes associated with other hematopoietic tumors as well as other cancers have been identified in PTCL, including SNF5 [16], LIN28B [17], MYC [18, 19], PI3K [18], mTOR [18], AKT [19, 20], MAF [21], genes involved in Notch signaling [22], members of the Polycomb repressive complex 2 including BMI1 [23] as well as genes regulating intrinsic [24] and extrinsic apoptosis [25].

One important approach towards increased knowledge about PTCL is through studies of genetically engineered mice, in which the impact of a number of genes has been investigated. Transgenic mice expressing ITK-SYK [26], Lin28b-transgenic mice [17], Snf5 deficient mice [16] as well as Tet2-knockdown mice [27] develop peripheral T-cell lymphoma-like diseases with variable latencies ranging from 11-67 weeks. For other disease-associated genes, including NPM-ALK [28, 29], Rho [30], Dnmt3a [31], STAT3 [32], Myc [33, 34], Akt [35], Maf, [21], Notch [36], Bmi1 [37] and Bcl-2 [38], it has been difficult to address their specific roles in PTCL development; as mice with genetic alterations involving these genes, again with prolonged lag times, frequently develop other hematologic malignancies, often of immature T cell origin, thereby masking their potential contribution to transformation of mature T cells.

Collectively, these studies indicate that although several disease-associated genes may contribute to the development of PTCL-like disease, the prolonged time preceding tumor development and the monoclonality of resulting tumors (where analyzed) in these experimental models indicate that additional, yet unidentified, genetic events were required for tumor development. Also, the notion that mature T cells may be resistant to oncogene driven transformation has been put forward [39]. An important, and so far, unanswered question is therefore if normal mature T cells can be tumor transformed, and in that case what would be the number and nature of driver events required. Herein, as a step towards an increased understanding of the cellular and molecular requirements for transformation, starting from a combinatorial *in vitro*-assay for T cell transformation, combinations of effector driver-genes sufficient for rapid, high frequency tumor transformation of mature murine T cells were identified.

## RESULTS

### Overexpression of distinct gene combinations results in T cell transformation

**Figure 1:**
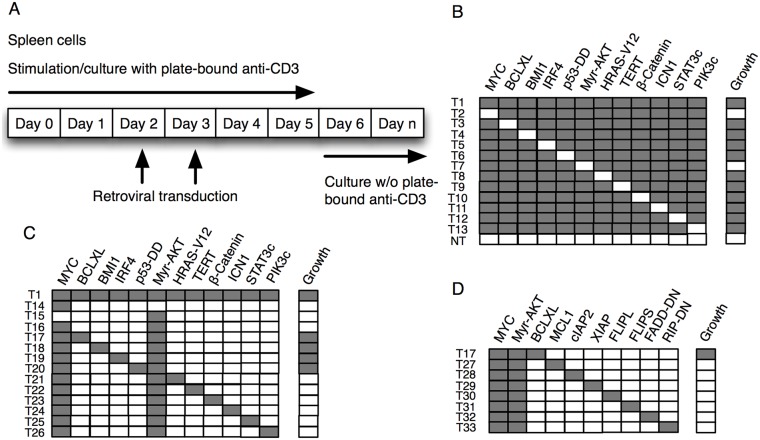
Transformation of mature T cells *in vitro* **(A)** Assay for T cell transformation. **(B-D)** Normal spleen cells from C57BL/6 mice were stimulated with plate bound anti-CD3 *in vitro* for two days, followed by transduction with combinations of retroviruses encoding (grey) or not (white) the indicated genes. Cultures were scored positive where growth could be recorded, through visual inspection, for more than 4 weeks.

Primary murine T cells were transduced at day 2 and 3 after anti-CD3 stimulation with a pool of 12 individual retroviruses encoding genes reported mutated or having altered expression patterns in PTCL and other tumors, namely *MYC*, *BCLXL*, *BMI1*, *IRF4*, dominant negative mutant *p53* (p53DD), constitutively active myristoylated *AKT* (Myr-AKT), constitutively active *β-catenin*, constitutively active *Notch* (ICN1), a constitutively activated form of *STAT3* (STAT3c) and a myristoylated constitutively active *PI3 kinase* (Myr-PIK3CA) as well as activated *HRAS* (HRAS-V12) and *hTERT*, followed by culture in the absence of anti-CD3 (Figure 1A–1B). As an initial criterion for potential transformation, expansive growth exceeding four weeks was chosen. Transduction of T cells with this pool of retroviruses reproducibly resulted in transformation (Figure 1B). Subsequently, one individual gene at a time was eliminated, followed by T cell transduction. Only in the pools lacking either MYC or Myr-AKT (AKT), did transformation not occur, indicating that MYC and AKT were both indispensable for transformation (Figure 1B). We subsequently tested pools of retroviruses containing *MYC* and *AKT* plus one additional construct. Four distinct combinations of genes leading to transformation were identified namely; *MYC+AKT+BCLXL* (reproduced > 15 times with cells from different mice and independent viral preparation), *MYC+AKT+BMI1* (reproduced > 5 times), *MYC+AKT+IRF4* (reproduced > 5 times) and *MYC+AKT+p53DD* (reproduced > 5 times) (Figure 1C). We tested if over-expression of other apoptotic inhibitors than BCLXL, including one additional members of the BCL2-family, MCL1, IAP-family members, cIAP2 and XIAP, inhibitors of the death receptor-mediated pathway of apoptosis, FLIP_L_ and FLIP_S_ as well as the dominant-negative mutants FADD-DN and RIP-DN, could cooperate with MYC and AKT in inducing T cell transformation, which was not the case (Figure 1D). It should be noted that absence of effects of some genes or gene combinations tested herein do not exclude their eventual importance during T-cell transformation but could reflect limitations in experimental design.

### Co-expression of MYC, AKT and BCLXL leads to rapid and high frequency-transformation of mature T cells

**Figure 2:**
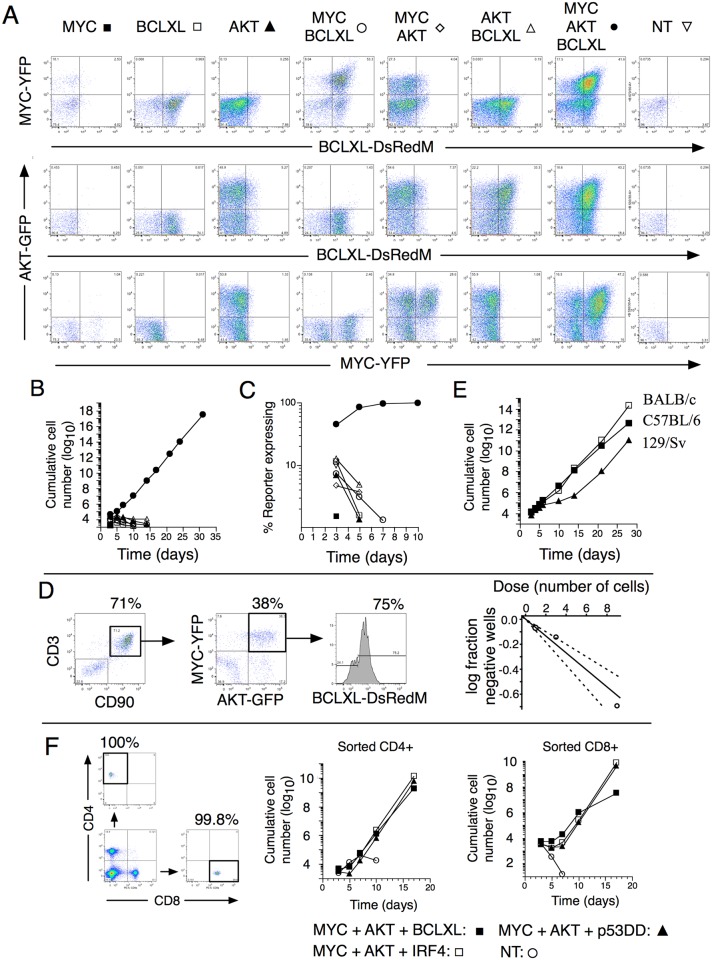
*In vitro*-transformation of mature T cells through co-expression of *MYC*, *AKT* and *BCLXL* **(A)** Reporter expression 3 days after retroviral transduction of spleen cells from C57BL/6 mice. **(B)** Total numbers of reporter expressing cells from cultures after transduction. **(C)** Proportions of reporter positive cells. **(D)** Expression of CD3, CD90, YFP, GFP and DsRed-monomer expression three days post- transduction, at the same day limiting dilution assay was set up. Cultures were scored positive when the cells could be expanded for prolonged periods of time. The graph shows fraction of negative cells versus original input number of cells per well. **(E)** Spleen cells from C57BL/6 (■), BALB/c (□) or 129/Sv (▲) mice were transduced with indicated retroviruses. Graph depicts cumulative cell numbers as a function of time. **(F)**. Spleen cells from C57BL/6 were sorted into CD4 and CD8 single positive cells (purity reanalysis shown) and stimulated with anti-CD3 and anti-CD28 followed by transduction with retroviruses encoding indicated gene combinations and cumulative cell numbers are shown.

As bicistronic vectors were used, MYC, AKT and BCLXL expression could be monitored through YFP, GFP and DsRed-monomer expression by flow cytometry (Figure 2A). Co-transduction of cells with MYC, AKT and BCLXL rapidly resulted in exponential growth, whereas expression of one or two of the genes in combination did not (Figure 2B). In cultures co-transduced with the three genes, cells co-expressing MYC, AKT and BCLXL rapidly outgrew other cells (Figure 2C). Limiting dilution assays were performed at day three after retroviral transduction. About 1 in 15 of total viable cells formed expandable clones (Figure 2D). Considering the number of input T cells co-expressing MYC, AKT and BCLXL (20%, Figure 2D), the corrected frequency of T cell transformation was 1 in 3 (1/2 −1/4, 95% confidence limits). Transformation by co-expression of MYC, AKT and BCLXL was observed in T cells from three out of three tested mouse strains (C57BL/6, BALB/c and 129/Sv) (Figure 2E). Plate-bound anti-CD3, exposure to ConA or allo-stimulation all resulted in transformation by co-expression of MYC, AKT and BCLXL (Supplementary Figure 1A), indicating that the mode of T cell activation did not seem to affect permissiveness for transformation.

Surface phenotypes of the transformed cells revealed a large degree of heterogeneity concerning expression of the T cell markers CD4, CD8, CD3 and CD90. Often, cells were double positive (DP) for CD4 and CD8 with variable expression of CD3. (Supplementary Figure 1A-B). The phenotypes of clones obtained in limiting dilution also showed variable phenotypes ranging from CD4 and CD8 single positive (SP) cells, DP and double negative (DN) cells (Supplementary Figure 2). To directly test if SP cells were targets of transformation, T cells were sorted into CD4 and CD8 SP cells (> 99% purity, Figure 2F) followed by transformation, resulting in rapid entry into exponential growth (Figure 2F). Phenotypic analyses clearly indicated that transformed cells started off as SP T cells but thereafter lost or gained expression of CD4, CD8 (Supplementary Figure 3), reminiscent of the variable expression of T-cell associated surface-receptors in human PTCL [40].

### Transformed T cells form aggressive tumors *in vivo*

**Figure 3:**
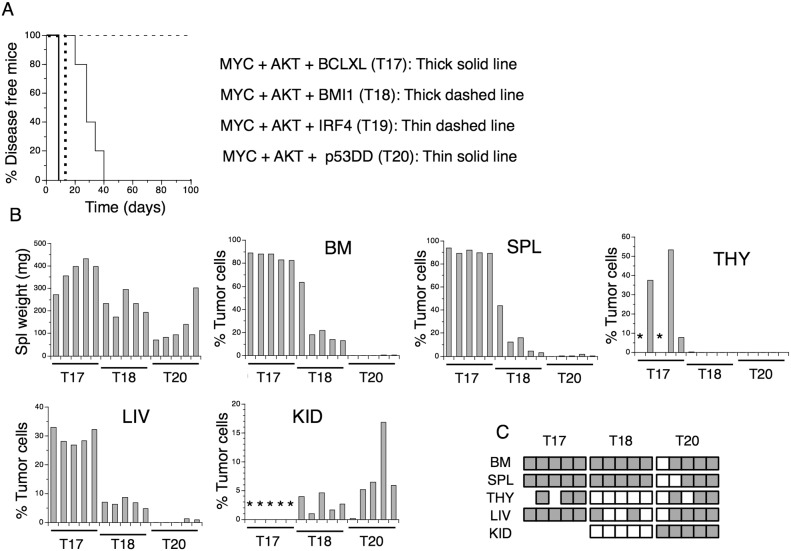
Transformed T cells form aggressive tumors *in vivo* **(A)** Kaplan-Meier plots of sub-lethally irradiated C57BL/6 mice (5 mice per group) injected with T cells transformed with indicated gene combinations **(B)** Spleen weight and % infiltrating (YFP^+^ GFP^+^ DsRed-monomer^+^) cells in bone marrow (BM), spleen (SPL), thymus (THY), liver (LIV) and kidney (KID) of mice injected with T17, T18 and T20 cells. ^*^indicate not analyzed. **(C)** Re-culture of cells from indicated organs of 5 individual mice injected with transformed cells. Grey square: YFP^+^ GFP^+^ DsRed-monomer^+^ tumor cell re-growth. White square: no tumor cell re-growth. No square: not tested.

In an initial experiment, injection of mice with T17 cells (MYC+AKT+BCLXL) resulted in death of 3 out of 4 animals already 12 days after injection, whereas mice injected with A20 cells (MYC+AKT+p53DD) were judged moribund at days 28-31. All mice injected with A20 cells had at least one enlarged kidney and flow cytometric analyzes revealed large infiltrations of tumor cells in kidneys but also to some extent in livers (Supplementary Figures 4, 5, Supplementary Table 1). Mice injected with independently derived T17 cells showed signs of aggressive disease and were considered moribund 9 days after injection and were euthanized (Figure 3A). The mice exhibited splenomegaly and massive numbers of tumor cells were detected in the bone marrow, spleen, liver and thymus (Figure 3B, Supplementary Figure 5 and Supplementary Table 2A). No signs of lymphadenopathy or infiltration in the kidneys were observed. Tumor cells from all analyzed organs grew out in secondary cultures *in vitro* (Figure 3C). Mice injected with T18 cells (MYC+AKT+BMI1), were euthanized at day 13, exhibited less pronounced splenomegaly and contained more modest numbers of tumor cells in the bone marrow, spleen and liver, accumulated tumor cells in the kidneys but not in the thymus (Figure 3B, Supplementary Table 2B). Mice injected with T20 cells (MYC+AKT+p53DD) from an independent experiment, were euthanized between days 20-40, displayed no signs of lymphadenopathy and splenomegaly was observed only in one out of five mice. Tumor cells were hardly detectable by flow cytometry, in bone marrow or spleen but livers and kidneys contained large numbers of tumor cells (Figure 3B, Supplementary Table 2C). Tumor cells, however, grew out from most organs in secondary cultures (Figure 3C), indicating their presence also in bone marrow and spleens. Surface phenotypes of the infiltrating tumor cells analyzed *ex vivo* and after re-culture *in vitro* are shown in Supplementary Figure 6A-6C. Findings using flow cytometry were supported by histological analyses indicating destruction of normal tissue architecture due to infiltration of tumor-like cells (Supplementary Figures 7-9). Unexpectedly, mice injected with T19 cells (MYC+AKT+IRF4) showed no signs of disease during the 100-day observation period (Figure 3A). Firm conclusions concerning the inability of T19 cells to form tumors *in vivo* would however require additional experiments.

### Expression of MYC, AKT and BCLXL is sufficient for tumor transformation of mature T cells

In the experiments above, cells were kept in culture during 8-12 weeks, prior to injection into mice. A possibility would be that rare cells, with potential additional genetic lesions required for tumor growth, could have been selected for during *in vitro* culture or after injection *in vivo*. As genetic aberrations are rare events, it would be expected that if additional genetics events, in conjunction with expression of MYC, AKT and BCLXL would be required for tumor growth *in vivo*, then this would result in monoclonal tumors, and conversely, if not, tumors would be polyclonal. T cells were transduced and around 3.5×10^5^ cells co- expressing markers for MYC, AKT and BCLCL (Supplementary Figure 10) were injected into mice already three days after transduction. Mice were euthanized 8 days after injection and bone marrow, spleen and liver contained very high levels of infiltrating tumor cells co-expressing the fluorescent reporters for MYC, AKT and BCLXL (Supplementary Table 3). Reporter-expressing T cells accounted for more than 90% of all T cells in all analyzed organs (Supplementary Table 3). Genomic DNA was prepared from spleens of three diseased animals and the T cell receptor beta variable (TcR-Vβ) genes were sequenced (Supplementary Table 4 and Dataset 1). The TcR-Vβ gene usage was similar between all sequenced samples giving an indication of a broad repertoire of tumor transformed T cells in the spleens of the three diseased mice (Supplementary Table 5).

**Figure 4:**
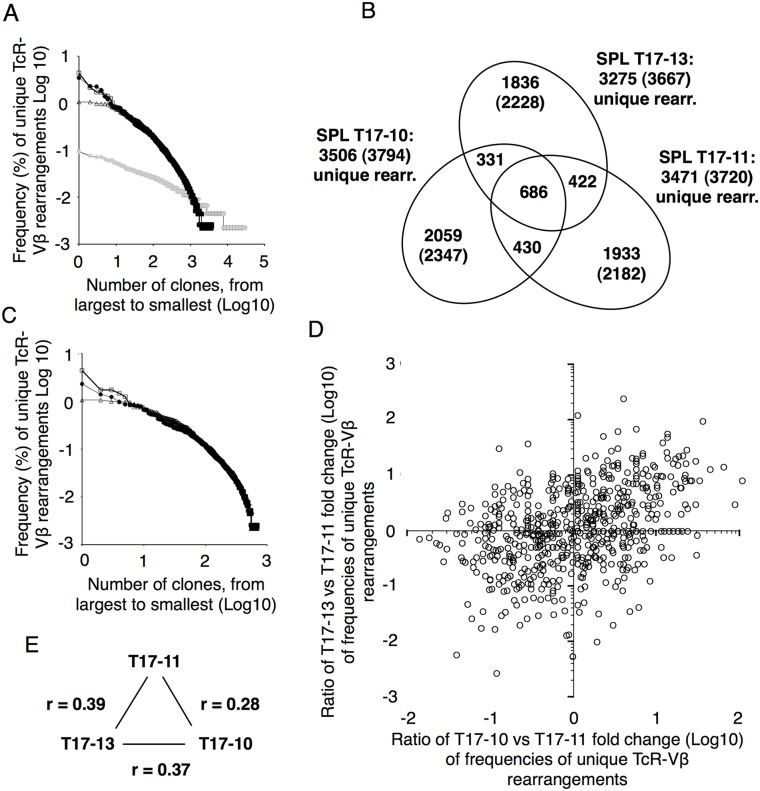
Tumor transformed T cells are polyclonal **(A)** Frequency distribution of unique TcR V-beta rearrangements in unstimulated spleen cells (open circles), stimulated and transduced cells prior to injection (grey circles) and in spleens of three diseased animals, T17-10 (open squares), T17-11 (filled circles) and T17-13 (open triangles). **(B)** Venn diagram showing overlap of unique TcR V-beta rearrangements in spleens of three individual animals. Unique TcR-beta rearrangements corresponding to endogenous rearrangements have been removed. Total numbers of unique TcR (including TcR V-beta rearrangements from potentially endogenous TcRs are indicated in parentheses. **(C)** Frequency distribution of unique TcR V-beta rearrangements in the 686 unique TcR V-beta rearrangements shared between T17-10, T17-11 and T17-13. **(D)** Fold change differences in frequency of the 686 common TcR V-beta rearrangements in SPL1 and SPL4 compared to SPL2. **(E)** Correlations of clone frequencies (Pearson correlation coefficient) of the 686 shared clones between.

Around 4000 unique productive TcR-Vβ gene rearrangements were identified in each spleen from the diseased animals (Supplementary Dataset 1). When subtracted for the number of TcR-Vβ gene rearrangements possibly corresponding to endogenous T cells, assuming these would have the smallest clone sizes, around 3500 unique productive TcR-Vβ gene rearrangements per mouse spleen were identified at this relatively low level of sequencing depth (Figure 4B). This indicates that the repertoire of tumor transformed T cells in this experimental setting is polyclonal in turn suggesting that genetic aberrations in addition to overexpression of MYC, AKT and BCLXL would not be required for tumor transformation of mature T cells.

However, a clear bias towards larger clone sizes could be observed in the spleens of the diseased mice as compared to normal spleen or anti-CD3 stimulated splenic T cells (Figure 4A), indicating clonal competition among tumor-transformed T cells. We therefore compared frequencies of 686 identical clones that were identified in all three mice (Figure 4B). Although the overall clone size distribution of the shared clones was similar (Figure 4C) individual clone sizes differed considerably among the three mice (Figure 4D, Supplementary Dataset 1), reflected in low Pearson correlation coefficients (Figure 4E), indicating that in this experimental setting, retroviral integration affecting either the expression levels of the transgenes or possibly affecting expression or function of neighboring genes, or the specificity of the TcR, did not to any larger extent influence the degree of clonal expansion of tumor-transformed T cells *in vivo*.

### Dependence of MYC, AKT and BCLXL expression for sustained proliferation and survival *in vitro*

**Figure 5:**
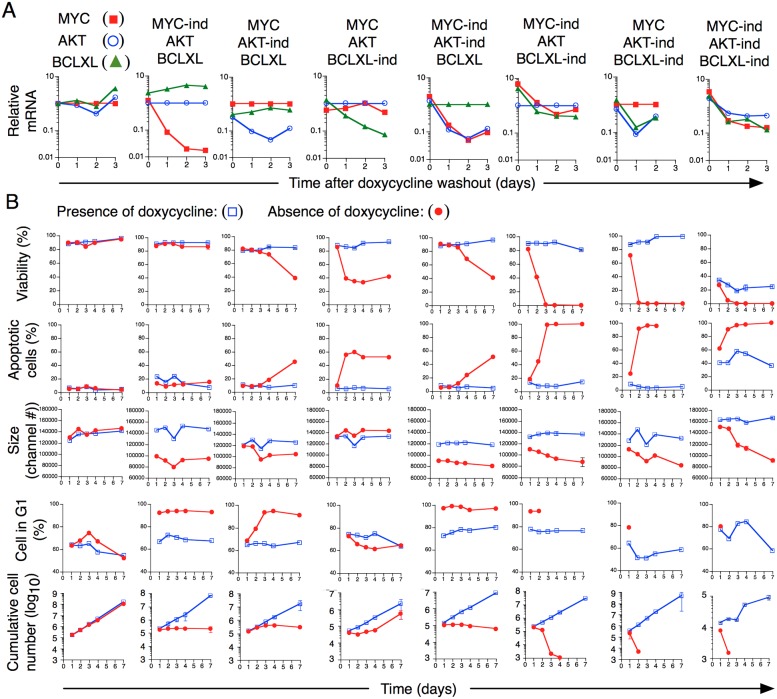
Dependence of expression on MYC, AKT and BCLXL for sustained growth of transformed T cells *in vitro* **(A)** Relative mRNA expression of MYC, AKT and BCLXL was quantified using real-time PCR on indicated days after removal of Dox. **(B)** Cellular viability, apoptosis, cell size, cell cycle status and cumulative number of cells were recorded for 7 days in the presence of absence of Dox.

In order to gain insight into the mechanistic roles of MYC, AKT and BCLXL in transformed cells, T cells with inducible expression (“Tet-on”) of MYC, AKT and BCLXL, or combinations thereof, were derived. Using real-time PCR we verified that MYC, AKT and BCLXL expression was downregulated as a consequence of doxycycline (Dox) washout (Figure 5A). Downregulation of MYC or AKT arrested cellular expansion, resulted in rapid accumulation of cells in the G_1/0_ phase of the cell cycle and a reduction in cell size, although this occurred with some delay after downregulation of AKT as compared to MYC. MYC downregulation was not associated with apoptotic cell death whereas apoptosis was detected after AKT downregulation, compatible with an anti-apoptotic effect of AKT [41]. (Figure 5B). BCLXL downregulation, as expected, led to induction of apoptotic cell death, paralleled by loss of cell viability but the around 60% fraction of apoptotic cells remained rather constant, from day 2 up till day 7. Despite the initial reduction in total numbers of live cells, between days 4 to 7, cell numbers increased exponentially to a similar rate as in cultures expressing BCLXL (Figure 5B). BCLXL downregulation resulted in a decreased proportion of (non-apoptotic) cells in G_1/0_ phase at least between days 1 to 4 (Figure 5B), in accordance with previous reports (reviewed in [42]). The reasons for the lower dependence on BCLXL expression in established cell lines as compared to during the initial transformation event (Figures 1–2) is unknown. Simultaneous downregulation of both MYC and AKT resulted in similar consequences as downregulation of MYC or AKT individually. Downregulation of MYC or AKT, together with downregulation of BCLXL however, resulted in massive apoptotic cell death with few, if any, live cells remaining 3 days after Dox washout. Similar data were obtained when simultaneously down-regulating all three genes (Figure 5B).

**Figure 6:**
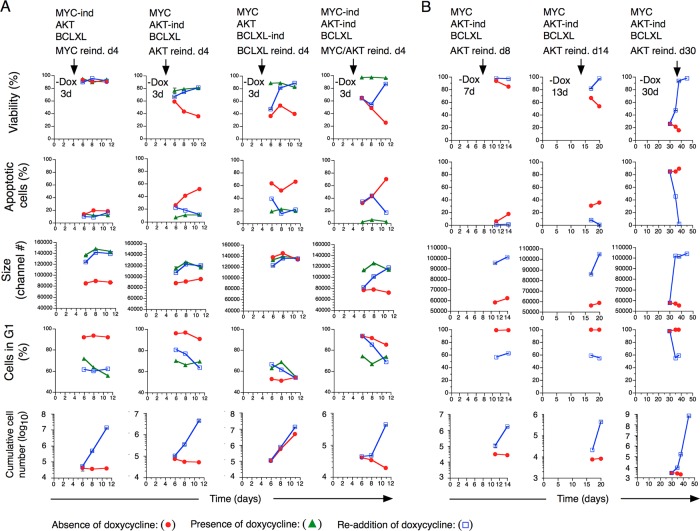
Consequences on transformed T cell growth characteristics after re-induction of MYC, AKT and BCLXL expression **(A)** Transformed T cell lines with indicated gene combinations were cultured in the presence or absence of Dox for 3 days followed by re-addition or not of Dox. Cellular viability, apoptosis, cell size, cell cycle status and cumulative number of cells were recorded at indicated days. **(B)** Transformed T cells with constitutive expression of MYC and BCLXL and inducible expression of AKT were cultured in the absence of Dox for 7, 13 and 30 days followed by Dox readdition at indicated time points.

Subsequently we investigated whether downregulation of MYC and/or AKT had resulted in induction of a reversible or irreversible state of cell cycle arrest, ie, quiescence or senescence. After 3 days in the absence of Dox after which the cells had entered a state of cell cycle arrest, Dox was re-added, which resulted in in re-induction of exponential growth, suggesting that downregulation of MYC and/or AKT resulted in induction of cellular quiescence (Figure 6A). Re-induction of BCLXL resulted in decreased frequency of apoptotic cells, increased viability although not affecting, the rate of cellular expansion (Figure 6A). We then analyzed consequences of prolonged periods of AKT downregulation (7, 13 and 30 days) before re-induction. As expected, cells rapidly accumulated in G_1/0_ accompanied by a gradual increase in the fraction of apoptotic cells, reaching 89%, 38 days after Dox washout. At all time points, re-induction of AKT resulted in reversal of cell cycle arrest, re-induction of exponential proliferation, increased cell size and decreased apoptotic rate (Figure 6B). Thus, our data would suggest that AKT in addition to counteracting quiescence induction also partially inhibited apoptotic cell death.

## DISCUSSION

Our results suggest that expression of MYC and activated AKT together with inhibition of intrinsic apoptosis via expression of BCLXL would constitute sufficient conditions for tumor transformation of mature mouse T cells, based on the rapid kinetics and high frequency of the process. This conclusion was further supported by the polyclonal repertoire of transformed T cells identified *in vivo* after injection into mice. Transformed T cells in this experimental setting could be considered malignant as they rapidly spread to different organs, resulted in destruction of normal tissue organization and leading to early death of the animals. To our knowledge, this is the first description of defined genetic factors sufficient for transformation of mature T cells. Our results do not support the notions that mature T cells would be resistant to oncogene transformation [39] unless occurring in a monoclonal T cell environment [43]. However, differences in experimental design as well as the genes tested, LMO2, TCL1, a constitutively active mutant of TrkA and NPL/ALK fusion protein, compared to those assayed herein, likely can explain the different results obtained.

As expression of BMI1 or dominant negative p53 could substitute for BCLXL, the main tumor suppressive effect of p53 that had to be inhibited in order for transformation to occur in mature T cells, likely consisted in inhibition of intrinsic apoptosis. In addition to promoting proliferation, pro-apoptotic effects of MYC are well documented and can at least partly be inhibited by expression of anti-apoptotic Bcl-2 family proteins reviewed in [44]. In accordance, co-expression of MYC together with BCLXL accelerated tumorigenesis in several experimental models [45, 46]. From these data, it could be anticipated that overexpression of MYC and BCLXL would be required and maybe sufficient, for T cell transformation. However, although we found that overexpression of MYC and BCLXL in mature T cells was required, transformation did not occur. Instead, we unexpectedly identified a concomitant requirement for expression of activated AKT in order for transformation to occur. Mechanistically, we found that AKT exerted a dual function as it inhibited induction of, and promoted exit from, cellular quiescence and also to some extent inhibited induction of intrinsic apoptosis, in accordance with previous reports [41, 47]. In established tumor cell lines, downregulation of AKT resulted in cellular quiescence accompanied by a rather modest rate of apoptotic cell death, up to 15% live cells remained after 35 days. Simultaneous downregulation of both AKT and BCLXL however, resulted in complete cellular eradication via apoptotic cell death within days. If these results could be extrapolated to human T-cell tumors (and maybe also other tumor types), then an effective treatment regimen, if tolerated, could consist in simultaneous inhibition of AKT or TcR-signaling and inhibition of anti-apoptotic Bcl-2 proteins. In line with this, synergy between inhibition of members of the anti-apoptotic Bcl-2 family and inhibition of PI3K/AKT/mTor signaling has recently been reported for human myeloid leukemia and diffuse large B-cell lymphoma cells [48, 49]. Elucidation of the exact molecular mechanisms underlying the interplay of MYC and AKT during T cell transformation, would warrant further studies and the cells described herein, with inducible expression of AKT, would be suitable for such an exploration. It is however challenging as MYC may be able to regulate up to a third of all genes and numerous AKT substrates have been described [50, 51].

Differences in requirements for tumor transformation of mouse and human cells have been described and it is therefore an open question if human T cells would be permissive for transformation with the same gene combinations as we here describe for mouse T cells [52]. Nevertheless, we believe our findings are of clinical relevance in PTCL as a number of mutated genes, including RHOA, CD28, LCK, FYN, PDK1, PIC3CA, PIK3R1, AKT1 [6, 7, 13, 53], as well as the fusion proteins NPM-ALK and ITK-SYK [4, 5, 26, 54, 55] either are part of, or can activate, the TcR/PI3K/AKT signaling pathway and our prediction is therefore that expression of at least some of these mutated genes would have the ability to substitute for activated AKT in the experimental system described herein. Moreover, deregulated expression of MYC [18, 19], members of the Polycomb repressive complex 2 including BMI1 [23] as well as genes regulating intrinsic apoptosis [24] has been reported in PTCL. These genes we would denote as “effector drivers”, able to directly affect cellular expansion as opposed to “diversity drivers”, genes mutated or with altered expression that contribute to genetic or epigenetic instability or alterations in splicing fidelity, exemplified by TET2, DNMT3A and IDH2, also commonly mutated in PTCL [8–10, 13], thereby generating a diverse and abnormal repertoire of cells with altered gene functions and/or expression levels, allowing for selection and expansion of a subset of cells with altered expression of one or several effector driver genes.

Despite the aggressive characteristics of PTCL, it is difficult to establish cell lines from PTCL-patients, reflected in the modest number of cell lines available [56]. This could indicate that in many cases, PTCL cells would not (yet) have acquired a state of autonomous growth, in contrast to the tumor-transformed T cells describe herein. An interesting possibility would be that some (epi)-genetic events leading to MYC or AKT activity or inhibition of p53 activity and/or intrinsic apoptosis in PTCL cells would be substituted for by receptor/ligand interactions taking place in the tumor microenvironment *in vivo*.

It is our belief that the description of sufficient conditions for tumor transformation of T cells provided herein as well as the experimental model for T cell transformation will provide a framework for further dissection of the mechanisms behind tumor transformation of hematopoietic cells in general and for PTCL in particular.

## MATERIALS AND METHODS

### Mice

C57BL/6, BALB/c or 129/Sv mice were obtained and bred at the Department of Microbiology, Tumor and Cell Biology, Karolinska Institutet animal facility. Where indicated, sub-lethally irradiated (600Rad) C57BL/6 mice were injected i.v. with 1-1.5 × 10^6^ cells. All procedures were performed with relevant ethical permission according to local and national guidelines.

### Stimulation of T cells and retroviral transduction

Retroviral particles were produced as described followed by concentration using through centrifugation at 6000 × g overnight at +4°C [57]. Splenic cells (1×10^6^/ml) were stimulated with plate-bound anti-CD3 antibody (clone 2C11), 2.5 μg/ml ConA (GE Health Care) or irradiated (1500Rad) allogeneic BALB/c splenic cells in complete RPMI medium [58]. Where indicated, T cells were sorted with a BD FACSAria sorter (BD Biosciences, San Jose, CA) into CD4^+^ and CD8^+^ cell populations, followed by stimulation with plate-bound anti-CD3 antibody in the presence of soluble anti-CD28 antibody. Retroviral transduction of around 5×10^5^ stimulated spleen cells was performed at day 2 and day 3 after stimulation using spin infection in the presence of 8 μg/ml polybrene (Sigma-Aldrich). Cells were replated in culture medium without stimuli at day 6 and growth was followed for more than 4 weeks. For cloning of cells using limiting dilution, 60 cultures for each dilution with syngeneic irradiated (2000 rad) thymocytes (2.5×10^6^/ml) as feeder cells were used. Clones were thereafter expanded to substantial numbers in the absence of feeder cells, followed by phenotypic analysis. Results were analyzed as described [59].

### Retroviral vectors

pMSCV-IRES-EGFP and pMSCV-IRES-EYFP have been described previously [60, 61]. For construction of pMSCV-IRES-EGFPZeo, EGFP was amplified from MSCV-IRES-GFP with junction markers for Zeo in 3’ forward primer; AATACCATGGTGAGCAAGGG and reverse primer; GGCACTGGTCAACTTGGCGTCCATGCCGAGAGTGATC. Zeo containing junction marker in 5’ was amplified by PCR from pTracer-SV40 (Life Technologies); forward primer; GATCACTCTCGGCATGGACGCCAAGTTGACCAGTGCC and reverse primer*;* ATAAGCGGCCGCTCAGTCCTGCTCCTCGG. Then, EGFP and Zeo fragments were mixed together in a PCR reaction using forward primer; AATACCATGGTGAGCAAGGG and reverse primer; ATAAGCGGCCGCTCAGTCCTGCTCCTCGG, followed by ligation into pMSCV-IRES-EGFP, previously cleaved with NotI and NcoI to remove EGFP.

The pMSCV-IRES-DsRed-Monomer was made by replacing EGFP with DsRed-Monomer amplified from pDsRed-Monomer-C1 (Clontech Laboratories, Moutain View, CA, USA) using TACCGGTCGCCACCATGGAC and ATTTATAGCGGCCGCTTTACTGGGAGCCGGAGTGGC. MYC was isolated by PCR from cDNA from muscle cells from the Human Multiple Tissue cDNA Panel 1 (Clontech Laboratories) using the primers; ACGTGAATTCCACCATGCCCCTCAACGTTAGCTTC and TACGTCTCGAGCTTACGCACAAGAGTTCCGTAG followed by ligation into the *EcoRI* and *XhoI* sites of pMSCV-IRES-EYFP to obtain the pMSCV-MYC-IRES-EYFP expression vector. pMSCV-BCLXL-IRES-DsRed-Monomer was obtained by subcloning of an *EcoRI* fragment containing human BCLXL from the pLXIN-BCLXL expression vector [58]. BMI1 was obtained from MGC (accession nr: BC011652) and PCR amplified using primers; ACGTCAATTGACCACCATGCATCGAACAACGAGAATC and TACGCTCGAGTCAACCAGAAGAAGTTGCTG followed by ligation into the EcoRI/XhoI site of pMSCV-IRES-EGFP. IRF4 was amplified from cDNA clone MGC:23069 using ACGTGAATTCACCACCATGAACCTGGAGGGCGGCGG andTGCATCTCGAGGGAATGGCGGATAGATCTGTGG followed by ligation into the EcoRI/XhoI site of pMSCV-IRES-EGFPZeo. A dominant negative p53 construct (p53DD) [62], a gift from M. Oren, was subcloned using EcoRI into pMSCV-IRES-EYFP, generating pMSCV-p53DD-IRES-EYFP. Myr-HA-AKT was PCR-amplified from 901 pLNCX-myr-HA-Akt1 (a gift from William Sellers (Addgene plasmid #9005), [63]) using forward primer; ACTGTGAATTCACCACCATGGGGTCTTCAAAATCTAAAC and reverse primer; TGCATCTCGAGTCAGGCCGTGCCGCTGGCCG followed by ligation into the EcoRI/XhoI site of pMSCV-IRES-EGFPZeo. HRAS-V12 was amplified from pBabe-Bleo-Ras (a gift from Lars-Gunnar Larsson) using ACTGTGAATTCACCACCATGACGGAATATAAGCTGGTG and TGCATCTCGAGTCAGGAGAGCACACACTTGC followed by ligation into the EcoRI/XhoI site of pMSCV-IRES-EGFPZeo. pBABE-puro-hTERT was a gift from Bob Weinberg (Addgene plasmid #1771) [64]. pMSCV-beta-catenin-IRES-EGFP was a gift from Tannishtha Reya (Addgene plasmid #14717, [65]). pMSCV-ICN1-IRES-EGFP was obtained through amplification of a fragment containing the human ICN1 from EF.hlCN1.Ubc.GFP (a gift from Linzhao Cheng (Addgene plasmid #17626, [66]) using forward primer; ACTGTGAATTCACCACCATGCGGCGGCAGCATGGCCAG and reverse primer; TGCATCTCGAGTTACTTGAACGCCTCCGGGATG and inserted into the EcoRI/XhoI site of pMSCV-IRES-EGFP. pMXs-Stat3-C was a gift from Shinya Yamanaka (Addgene plasmid # 13373) [67]. pMSCV-Myr-HA-PIK3CA-IRES-EGFPZeo (Myr-HA-PIK3CA was PCR amplified from pBabe-puro-Myr-HA-PIK3CA, a gift from Jean Zhao (Addgene plasmid #12523) [68]) followed by ligation into the XhoI site of pMSCV-IRES-EGFPZeo using forward primer; TGCATCTCGAGTCAGTTCAATGCATGCTG and reverse primer; TGCATCTCGAGTCAGTTCAATGCATGCTG. Human MCL1 from pTOPO-MCL1, a gift from Ulrich Maurer (Addgene plasmid #21605) [69], was PCR amplified using primers; ACTGTGAATTCACCACCATGTTTGGCCTCAAAAGAAACGCG and TGCAGTCGACCTATCTTATTAGATATGCCAAACCAGCTCC followed by ligation into the EcoRI/XhoI site of pMSCV-IRES-EGFPZeo. Human cIAP-2 was excised from Flag-cIAP2/RK5 (a gift from Xiaolu Yang (Addgene plasmid #27973) [70] and cloned into the EcoRI site of pMSCV-IRES-EGFP. XIAP was amplified with CGCGAATTCATGACTTTTAACAGTTTTG and CGCCTCGAGCTATAGAGTTAGATTAAGAC from pEBB-XIAP (a gift from Jon Ashwell (Addgene plasmid #11558) [71] and inserted into the EcoRI/XhoI site of pMSCV-IRES-EGFP. pMSCV-FLIP_L_-IRES-EGFP and pMSCV-FLIP_S_-IRES-EGFP [58], as well as pMSCV-RIPDN-IRES-EGFP [60] have been described previously. FADD-DD was excised from NFD4-pcDNA3 using KpnI and XhoI and ligated into the EcoRI/XhoI site of pMSCV-IRES-EGFP. The region containing cPPT-TRE-mSEAP-PKG-rtTA2 was amplified from pTMPrtTA, a gift from Antonia Zanta Boussif [72], by PCR using the primers; ATATTAGATCTACTTTTAAAAGAAAAGGGGGGAT and TAAAAGTACTCGGACCGTCGTTACCCGGGGAGCA and subsequently ligated into pSIREN-RetroQ-ZsGreen (Clontech) from which the human U6 promoter and ZsGreen were removed using BglII and EcoRV. This vector (pSIR-TRE-mSEAP-PKG-rtTA2) was subsequently opened with MluI and NotI into which IRES-EGFP was inserted resulting in the pSIR-TRE-IRES-EGFP-PGK1-rtTA2. IRES-EGFP was amplified from pMSCV-IRES-EGFP with the primers TTTAACGCGTTTTAACCGGTGGATCAATTCCGCCCCT and AGAGTCGCGGCCGCT. This vector contains MluI and AgeI restriction enzyme recognition sites allowing for directional cloning of a gene-of-interest driven by the inducible promoter. MYC was amplified from pMSCV-MYC-IRES-EYFP with ACTCGTACGTACGCGTCACCATGCCCCTCAACGTTAGCTTC and TGCATACCGGTTTACGCACAAGAGTTCCGTAG; BCLXL from pMSCV-BCLXL-IRES-EGFP using ACGTACGCGTACCACCATGTCTCAGAGCAACCGGGAGCTG and TACGTACCGGTTCATTTCCGACTGAAGAGTGAGCCCAG and Myr-HA-AKT was PCR-amplified from pMSCV-Myr-HA-AKT-IRES-EGFPZeo using ACGTACGCGTACCACCATGGGGTCTTCAAAATCTAAACCAA and TGCATCACCGGTTCAGGCCGTGCCGCTGGCCG followed by ligation into pSIR-TRE-IRES-EGFP-PGK1-rtTA2. All plasmids used will be made available through Addgene.

### Flow cytometry

Cells from *in vitro* cultures or single cell suspensions obtained from organs from euthanized mice were stained with mAbs: anti-mouse CD4, anti-mouse CD8, anti-mouse CD3 and anti-mouse CD90 (BD Biosciences Pharmingen, San Diego, CA). Propidium iodide was used to evaluate viability and to eliminate dead cells from phenotypic analyses. Mean fluorescence intensity in the forward scatter channel (FSC) was used to estimate relative cell size. Cell cycle analysis was performed as described [73]. Cells were analyzed on a BD LSRFortessa Cell Analyzer (BD Biosciences) and the data analyzed with FlowJo software (Tree Star, Inc., San Carlos, CA). EGFP and EYFP signals were separated with a 526 nm dichroic mirror and 510/20 nm and 550/30 nm bandpass filters. Where indicated, numbers of viable cells were determined by cell counting using a BD LSRFortessa Cell Analyzer connected to a BD High Throughput Sampler (BD Biosciences). The number of live cells in 100 μl of cell suspension was determined in triplicates.

### RNA isolation and real-time PCR

Total RNA was isolated followed by synthesis of cDNA. Real-time PCR was carried out using TaqMan gene expression pre-synthesized reagents directed against *MYC*, *AKT* and *BCLXL* and *Gapdh* using a master mix in a 7500 Real-Time PCR system (Applied Biosystems, Foster City, CA). Relative gene expression was calculated using the ΔΔCt method.

### High throughput TcR V-beta deep sequencing

Genomic DNA was prepared using DNEasy minicolumns (Qiagen). Sequencing was performed by Adaptive Biotechnologies (Seattle, WA) using the Immunoseq platform and data were analyzed using the ImmunoSEQ Analyser software (Adaptive biotechnologies).

Supplementary Information accompanies this paper.

## SUPPLEMENTARY MATERIALS FIGURES AND TABLES





## References

[1] Inghirami G, Chan WC, Pileri S (2015). Peripheral T-cell and NK cell lymphoproliferative disorders: cell of origin, clinical and pathological implications. Immunol Rev.

[2] Swerdlow SH, Campo E, Pileri SA, Harris NL, Stein H, Siebert R, Advani R, Ghielmini M, Salles GA, Zelenetz AD, Jaffe ES (2016). The 2016 revision of the World Health Organization classification of lymphoid neoplasms. Blood.

[3] Vose J, Armitage J, Weisenburger D (2008). International peripheral T-cell and natural killer/t-cell lymphoma study: pathology findings and clinical outcomes. J Clin Oncol.

[4] Morris SW, Kirstein MN, Valentine MB, Dittmer KG, Shapiro DN, Saltman DL, Look AT (1994). Fusion of a kinase gene, ALK, to a nucleolar protein gene, NPM, in non-Hodgkin’s lymphoma. Science.

[5] Streubel B, Vinatzer U, Willheim M, Raderer M, Chott A (2006). Novel t(5;9)(q33;q22) fuses ITK to SYK in unspecified peripheral T-cell lymphoma. Leukemia.

[6] Sakata-Yanagimoto M, Enami T, Yoshida K, Shiraishi Y, Ishii R, Miyake Y, Muto H, Tsuyama N, Sato-Otsubo A, Okuno Y, Sakata S, Kamada Y, Nakamoto-Matsubara R (2014). Somatic RHOA mutation in angioimmunoblastic T cell lymphoma. Nat Genet.

[7] Palomero T, Couronne L, Khiabanian H, Kim MY, Ambesi-Impiombato A, Perez-Garcia A, Carpenter Z, Abate F, Allegretta M, Haydu JE, Jiang X, Lossos IS, Nicolas C (2014). Recurrent mutations in epigenetic regulators, RHOA and FYN kinase in peripheral T cell lymphomas. Nat Genet.

[8] Quivoron C, Couronne L, Della Valle V, Lopez CK, Plo I, Wagner-Ballon O, Do Cruzeiro M, Delhommeau F, Arnulf B, Stern MH, Godley L, Opolon P, Tilly H (2011). TET2 inactivation results in pleiotropic hematopoietic abnormalities in mouse and is a recurrent event during human lymphomagenesis. Cancer Cell.

[9] Couronne L, Bastard C, Bernard OA (2012). TET2 and DNMT3A mutations in human T-cell lymphoma. N Engl J Med.

[10] Cairns RA, Iqbal J, Lemonnier F, Kucuk C, de Leval L, Jais JP, Parrens M, Martin A, Xerri L, Brousset P, Chan LC, Chan WC, Gaulard P (2012). IDH2 mutations are frequent in angioimmunoblastic T-cell lymphoma. Blood.

[11] Abdel-Wahab O, Levine RL (2013). Mutations in epigenetic modifiers in the pathogenesis and therapy of acute myeloid leukemia. Blood.

[12] The Cancer Genome Atlas Network (2013). Genomic and epigenomic landscapes of adult de novo acute myeloid leukemia. N Engl J Med.

[13] Vallois D, Dobay MP, Morin RD, Lemonnier F, Missiaglia E, Juilland M, Iwaszkiewicz J, Fataccioli V, Bisig B, Roberti A, Grewal J, Bruneau J, Fabiani B (2016). Activating mutations in genes related to TCR signaling in angioimmunoblastic and other follicular helper T-cell-derived lymphomas. Blood.

[14] Crescenzo R, Abate F, Lasorsa E, Tabbo F, Gaudiano M, Chiesa N, Di Giacomo F, Spaccarotella E, Barbarossa L, Ercole E, Todaro M, Boi M, Acquaviva A (2015). Convergent mutations and kinase fusions lead to oncogenic STAT3 activation in anaplastic large cell lymphoma. Cancer Cell.

[15] Feldman AL, Law M, Remstein ED, Macon WR, Erickson LA, Grogg KL, Kurtin PJ, Dogan A (2009). Recurrent translocations involving the IRF4 oncogene locus in peripheral T-cell lymphomas. Leukemia.

[16] Wang X, Werneck MB, Wilson BG, Kim HJ, Kluk MJ, Thom CS, Wischhusen JW, Evans JA, Jesneck JL, Nguyen P, Sansam CG, Cantor H, Roberts CW (2011). TCR-dependent transformation of mature memory phenotype T cells in mice. J Clin Invest.

[17] Beachy SH, Onozawa M, Chung YJ, Slape C, Bilke S, Francis P, Pineda M, Walker RL, Meltzer P, Aplan PD (2012). Enforced expression of Lin28b leads to impaired T-cell development, release of inflammatory cytokines, and peripheral T-cell lymphoma. Blood.

[18] Iqbal J, Wright G, Wang C, Rosenwald A, Gascoyne RD, Weisenburger DD, Greiner TC, Smith L, Guo S, Wilcox RA, Teh BT, Lim ST, Tan SY (2014). Gene expression signatures delineate biological and prognostic subgroups in peripheral T-cell lymphoma. Blood.

[19] Warner K, Crispatzu G, Al-Ghaili N, Weit N, Florou V, You MJ, Newrzela S, Herling M (2013). Models for mature T-cell lymphomas--a critical appraisal of experimental systems and their contribution to current T-cell tumorigenic concepts. Crit Rev Oncol Hematol.

[20] Cai Q, Deng H, Xie D, Lin T, Lin T (2012). Phosphorylated AKT protein is overexpressed in human peripheral T-cell lymphomas and predicts decreased patient survival. Clin Lymphoma Myeloma Leuk.

[21] Morito N, Yoh K, Fujioka Y, Nakano T, Shimohata H, Hashimoto Y, Yamada A, Maeda A, Matsuno F, Hata H, Suzuki A, Imagawa S, Mitsuya H (2006). Overexpression of c-Maf contributes to T-cell lymphoma in both mice and human. Cancer Res.

[22] Kamstrup MR, Biskup E, Gjerdrum LM, Ralfkiaer E, Niazi O, Gniadecki R (2014). The importance of Notch signaling in peripheral T-cell lymphomas. Leuk Lymphoma.

[23] Kim SH, Yang WI, Min YH, Ko YH, Yoon SO (2016). The role of the polycomb repressive complex pathway in T and NK cell lymphoma: biological and prognostic implications. Tumour Biol.

[24] Rassidakis GZ, Jones D, Lai R, Ramalingam P, Sarris AH, McDonnell TJ, Medeiros LJ (2003). BCL-2 family proteins in peripheral t-cell lymphomas: correlation with tumour apoptosis and proliferation. J Pathol.

[25] Oyarzo MP, Medeiros LJ, Atwell C, Feretzaki M, Leventaki V, Drakos E, Amin HM, Rassidakis GZ (2006). c-FLIP confers resistance to FAS-mediated apoptosis in anaplastic large-cell lymphoma. Blood.

[26] Pechloff K, Holch J, Ferch U, Schweneker M, Brunner K, Kremer M, Sparwasser T, Quintanilla-Martinez L, Zimber-Strobl U, Streubel B, Gewies A, Peschel C, Ruland J (2010). The fusion kinase ITK-SYK mimics a T cell receptor signal and drives oncogenesis in conditional mouse models of peripheral T cell lymphoma. J Exp Med.

[27] Muto H, Sakata-Yanagimoto M, Nagae G, Shiozawa Y, Miyake Y, Yoshida K, Enami T, Kamada Y, Kato T, Uchida K, Nanmoku T, Obara N, Suzukawa K (2014). Reduced TET2 function leads to T-cell lymphoma with follicular helper T-cell-like features in mice. Blood Cancer J.

[28] Chiarle R, Gong JZ, Guasparri I, Pesci A, Cai J, Liu J, Simmons WJ, Dhall G, Howes J, Piva R, Inghirami G (2003). NPM-ALK transgenic mice spontaneously develop T-cell lymphomas and plasma cell tumors. Blood.

[29] Jager R, Hahne J, Jacob A, Egert A, Schenkel J, Wernert N, Schorle H, Wellmann A (2005). Mice transgenic for NPM-ALK develop non-hodgkin lymphomas. Anticancer Res.

[30] Cleverley SC, Costello PS, Henning SW, Cantrell DA (2000). Loss of Rho function in the thymus is accompanied by the development of thymic lymphoma. Oncogene.

[31] Mayle A, Yang L, Rodriguez B, Zhou T, Chang E, Curry CV, Challen GA, Li W, Wheeler D, Rebel VI, Goodell MA (2015). Dnmt3a loss predisposes murine hematopoietic stem cells to malignant transformation. Blood.

[32] Couronne L, Scourzic L, Pilati C, Della Valle V, Duffourd Y, Solary E, Vainchenker W, Merlio JP, Beylot-Barry M, Damm F, Stern MH, Gaulard P, Lamant L (2013). STAT3 mutations identified in human hematologic neoplasms induce myeloid malignancies in a mouse bone marrow transplantation model. Haematologica.

[33] Smith DP, Bath ML, Metcalf D, Harris AW, Cory S (2006). MYC levels govern hematopoietic tumor type and latency in transgenic mice. Blood.

[34] Stewart M, Cameron E, Campbell M, McFarlane R, Toth S, Lang K, Onions D, Neil JC (1993). Conditional expression and oncogenicity of c-myc linked to a CD2 gene dominant control region. Int J Cancer.

[35] Malstrom S, Tili E, Kappes D, Ceci JD, Tsichlis PN (2001). Tumor induction by an Lck-MyrAkt transgene is delayed by mechanisms controlling the size of the thymus. Proc Natl Acad Sci U S A.

[36] Pear WS, Aster JC, Scott ML, Hasserjian RP, Soffer B, Sklar J, Baltimore D (1996). Exclusive development of T cell neoplasms in mice transplanted with bone marrow expressing activated notch alleles. J Exp Med.

[37] Haupt Y, Bath ML, Harris AW, Adams JM (1993). Bmi-1 transgene induces lymphomas and collaborates with myc in tumorigenesis. Oncogene.

[38] Linette GP, Hess JL, Sentman CL, Korsmeyer SJ (1995). Peripheral T-cell lymphoma in lckpr-bcl-2 transgenic mice. Blood.

[39] Newrzela S, Cornils K, Li Z, Baum C, Brugman MH, Hartmann M, Meyer J, Hartmann S, Hansmann ML, Fehse B, von Laer D (2008). Resistance of mature T cells to oncogene transformation. Blood.

[40] Agostinelli C, Piccaluga PP, Went P, Rossi M, Gazzola A, Righi S, Sista T, Campidelli C, Zinzani PL, Falini B, Pileri SA (2008). Peripheral T cell lymphoma, not otherwise specified: the stuff of genes, dreams and therapies. J Clin Pathol.

[41] Kennedy SG, Wagner AJ, Conzen SD, Jordan J, Bellacosa A, Tsichlis PN, Hay N (1997). The PI 3-kinase/Akt signaling pathway delivers an anti-apoptotic signal. Genes Dev.

[42] Zinkel S, Gross A, Yang E (2006). BCL2 family in DNA damage and cell cycle control. Cell Death Differ.

[43] Newrzela S, Al-Ghaili N, Heinrich T, Petkova M, Hartmann S, Rengstl B, Kumar A, Jack HM, Gerdes S, Roeder I, Hansmann ML, von Laer D (2012). T-cell receptor diversity prevents T-cell lymphoma development. Leukemia.

[44] McMahon SB (2014). MYC and the control of apoptosis. Cold Spring Harb Perspect Med.

[45] Strasser A, Harris AW, Bath ML, Cory S (1990). Novel primitive lymphoid tumours induced in transgenic mice by cooperation between myc and bcl-2. Nature.

[46] Swanson PJ, Kuslak SL, Fang W, Tze L, Gaffney P, Selby S, Hippen KL, Nunez G, Sidman CL, Behrens TW (2004). Fatal acute lymphoblastic leukemia in mice transgenic for B cell-restricted bcl-xl and c-myc. J Immunol.

[47] Liang J, Slingerland JM (2003). Multiple roles of the PI3k/PKB (Akt) pathway in cell cycle progression. Cell Cycle.

[48] Choudhary GS, Al-Harbi S, Mazumder S, Hill BT, Smith MR, Bodo J, Hsi ED, Almasan A (2015). MCL-1 and BCL-xL-dependent resistance to the BCL-2 inhibitor abt-199 can be overcome by preventing PI3K/AKT/mTOR activation in lymphoid malignancies. Cell Death Dis.

[49] Rahmani M, Aust MM, Attkisson E, Williams DC, Ferreira-Gonzalez A, Grant S (2013). Dual inhibition of Bcl-2 and Bcl-xL strikingly enhances PI3K inhibition-induced apoptosis in human myeloid leukemia cells through a GSK3- and Bim-dependent mechanism. Cancer Res.

[50] Dang CV (2012). MYC on the path to cancer. Cell.

[51] Vivanco I, Sawyers CL (2002). The phosphatidylinositol 3-Kinase AKT pathway in human cancer. Nat Rev Cancer.

[52] Rangarajan A, Weinberg RA (2003). Opinion: comparative biology of mouse versus human cells: modelling human cancer in mice. Nat Rev Cancer.

[53] Saoudi A, Kassem S, Dejean A, Gaud G (2014). Rho-GTPases as key regulators of T lymphocyte biology. Small GTPases.

[54] Bai RY, Ouyang T, Miething C, Morris SW, Peschel C, Duyster J (2000). Nucleophosmin-anaplastic lymphoma kinase associated with anaplastic large-cell lymphoma activates the phosphatidylinositol 3-kinase/Akt antiapoptotic signaling pathway. Blood.

[55] Slupianek A, Nieborowska-Skorska M, Hoser G, Morrione A, Majewski M, Xue L, Morris SW, Wasik MA, Skorski T (2001). Role of phosphatidylinositol 3-kinase-Akt pathway in nucleophosmin/anaplastic lymphoma kinase-mediated lymphomagenesis. Cancer Res.

[56] Drexler HG (2010). Guide to leukemia-lymphoma cell lines.

[57] Högstrand K, Hejll E, Sander B, Rozell B, Larsson LG, Grandien A (2012). Inhibition of the intrinsic but not the extrinsic apoptosis pathway accelerates and drives MYC-driven tumorigenesis towards acute myeloid leukemia. PLoS One.

[58] Djerbi M, Darreh-Shori T, Zhivotovsky B, Grandien A (2001). Characterization of the human FLICE-inhibitory protein locus and comparison of the anti-apoptotic activity of four different flip isoforms. Scand J Immunol.

[59] Hu Y, Smyth GK (2009). ELDA: extreme limiting dilution analysis for comparing depleted and enriched populations in stem cell and other assays. J Immunol Methods.

[60] Djerbi M, Malinowski MM, Yagita H, Zhivotovsky B, Grandien A (2007). Participation of FLIP, RIP and Bcl-x(L) in fas-mediated T-cell death. Scand J Immunol.

[61] Persons DA, Allay JA, Allay ER, Smeyne RJ, Ashmun RA, Sorrentino BP, Nienhuis AW (1997). Retroviral-mediated transfer of the green fluorescent protein gene into murine hematopoietic cells facilitates scoring and selection of transduced progenitors *in vitro* and identification of genetically modified cells *in vivo*. Blood.

[62] Shaulian E, Zauberman A, Ginsberg D, Oren M (1992). Identification of a minimal transforming domain of p53: negative dominance through abrogation of sequence-specific DNA binding. Mol Cell Biol.

[63] Ramaswamy S, Nakamura N, Vazquez F, Batt DB, Perera S, Roberts TM, Sellers WR (1999). Regulation of G1 progression by the PTEN tumor suppressor protein is linked to inhibition of the phosphatidylinositol 3-kinase/Akt pathway. Proc Natl Acad Sci U S A.

[64] Counter CM, Hahn WC, Wei W, Caddle SD, Beijersbergen RL, Lansdorp PM, Sedivy JM, Weinberg RA (1998). Dissociation among *in vitro* telomerase activity, telomere maintenance, and cellular immortalization. Proc Natl Acad Sci U S A.

[65] Reya T, Duncan AW, Ailles L, Domen J, Scherer DC, Willert K, Hintz L, Nusse R, Weissman IL (2003). A role for Wnt signalling in self-renewal of haematopoietic stem cells. Nature.

[66] Yu X, Alder JK, Chun JH, Friedman AD, Heimfeld S, Cheng L, Civin CI (2006). HES1 inhibits cycling of hematopoietic progenitor cells via DNA binding. Stem Cells.

[67] Takahashi K, Yamanaka S (2006). Induction of pluripotent stem cells from mouse embryonic and adult fibroblast cultures by defined factors. Cell.

[68] Zhao JJ, Liu Z, Wang L, Shin E, Loda MF, Roberts TM (2005). The oncogenic properties of mutant p110alpha and p110beta phosphatidylinositol 3-kinases in human mammary epithelial cells. Proc Natl Acad Sci U S A.

[69] Maurer U, Charvet C, Wagman AS, Dejardin E, Green DR (2006). Glycogen synthase kinase-3 regulates mitochondrial outer membrane permeabilization and apoptosis by destabilization of MCL-1. Mol Cell.

[70] Hu S, Du MQ, Park SM, Alcivar A, Qu L, Gupta S, Tang J, Baens M, Ye H, Lee TH, Marynen P, Riley JL, Yang X (2006). cIAP2 is a ubiquitin protein ligase for BCL10 and is dysregulated in mucosa-associated lymphoid tissue lymphomas. J Clin Invest.

[71] Yang Y, Fang S, Jensen JP, Weissman AM, Ashwell JD (2000). Ubiquitin protein ligase activity of IAPs and their degradation in proteasomes in response to apoptotic stimuli. Science.

[72] Barde I, Zanta-Boussif MA, Paisant S, Leboeuf M, Rameau P, Delenda C, Danos O (2006). Efficient control of gene expression in the hematopoietic system using a single tet-on inducible lentiviral vector. Mol Ther.

[73] Riccardi C, Nicoletti I (2006). Analysis of apoptosis by propidium iodide staining and flow cytometry. Nat Protoc.

