# Supplementary material for: Transformation of mouse T cells requires MYC and AKT activity in conjunction with inhibition of intrinsic apoptosis
Source: Oncotarget. 2018 Apr 20;9(30):21396–410. doi: 10.18632/oncotarget.25113 (PMC5940390; doi:10.18632/oncotarget.25113)
Supplement: Supplementary file 1 [file oncotarget-09-21396-s001.pdf]

Transformation of mouse T cells requires MYC and AKT activity in conjunction with inhibition of intrinsic apoptosis

SUPPLEMENTARY MATERIALS

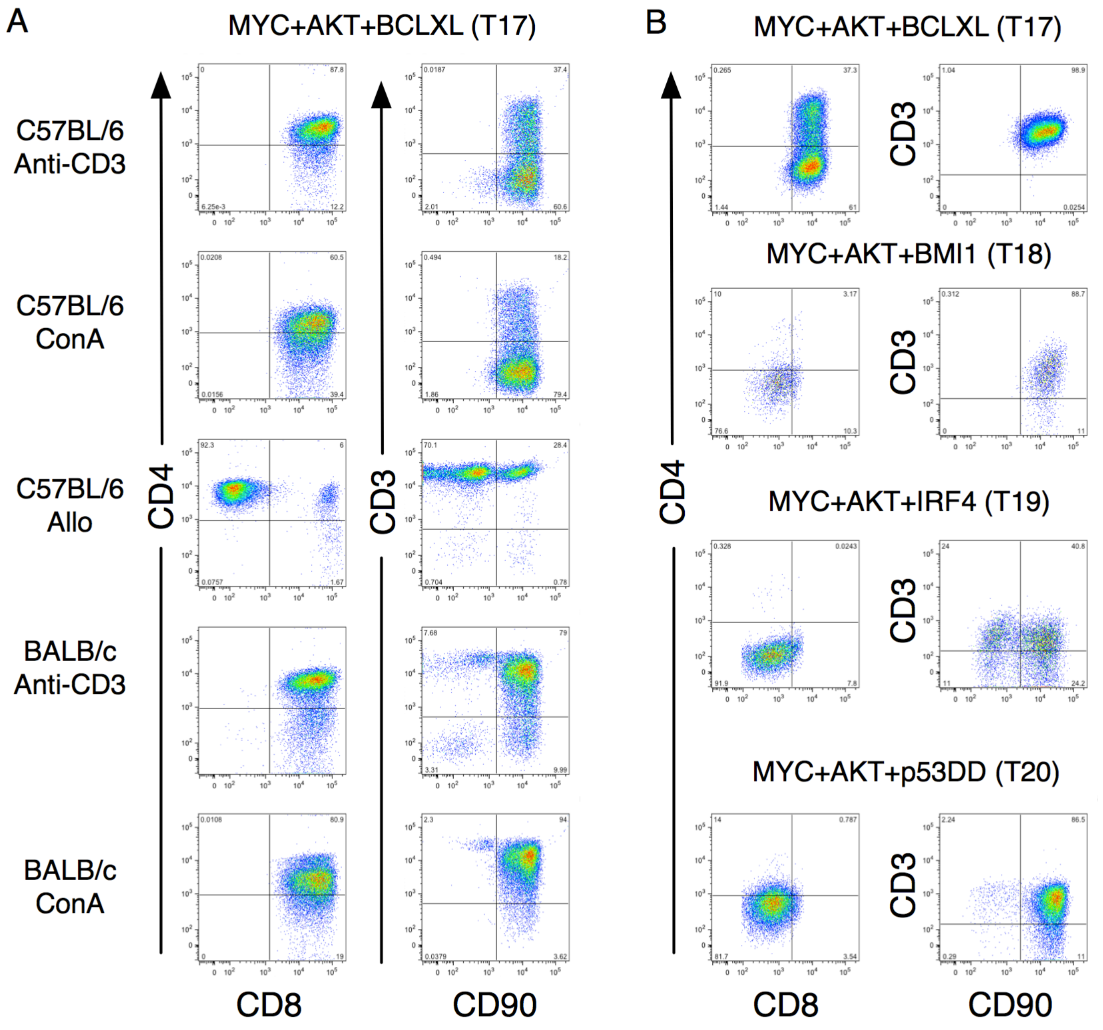

Supplementary Figure 1: Phenotypes of transformed cells using different stimuli, mouse strains and gene combinations. (A) Normal spleen cells from C57BL/6 or BALB/c mice were stimulated with plate bound anti-CD3 mAbs or ConA. Spleen cells from C57BL/6 were allo-stimulated with irradiated spleen cells from BALB/c *in vitro* and transduced with retroviruses encoding *MYC, AKT* and *BCLXL* (T17). 4 to 8 weeks later, cells were stained with antibodies against CD4, CD8, CD3 and CD90 and analyzed with flow cytometry. **(B)** Anti-CD3 stimulated spleen cells from C57BL/6 were transduced with 4 different gene combinations of retroviral vectors as indicated. Phenotypic analyses were performed as in (A).

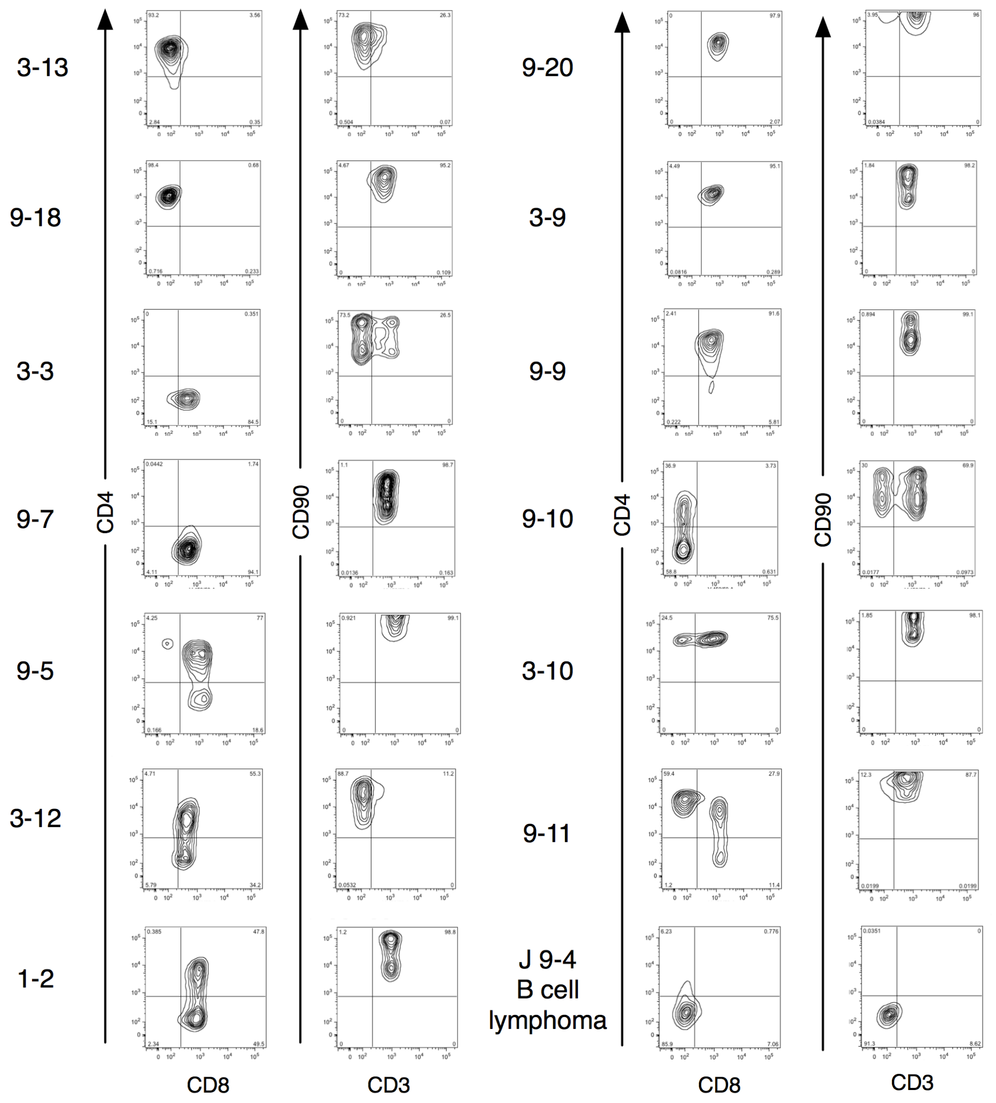

Supplementary Figure 2: Phenotype of clones of transformed T cells obtained through transduction with retroviruses encoding MYC, AKT and BCLXL. Surface expression of CD4, CD8, CD3 and CD90 in 13 cloned transformed T cells. A B cell lymphoma, J 9-4, is shown as negative control. In a total of 26 analyzed clones, 3 were mainly single positive CD4+, 4 single positive CD8+, 10 consisted of cells being both CD8+ and CD4, CD8 double positive, 4 CD4, CD8 double negative. One clone consisted of both CD4 single positive and CD3 and CD8 double positive cells and one clone consisted of cells being single positive for CD4 and CD8 and CD4, CD8 double positive cells. All clones were positive for GFP, YFP and DsRed-monomer.

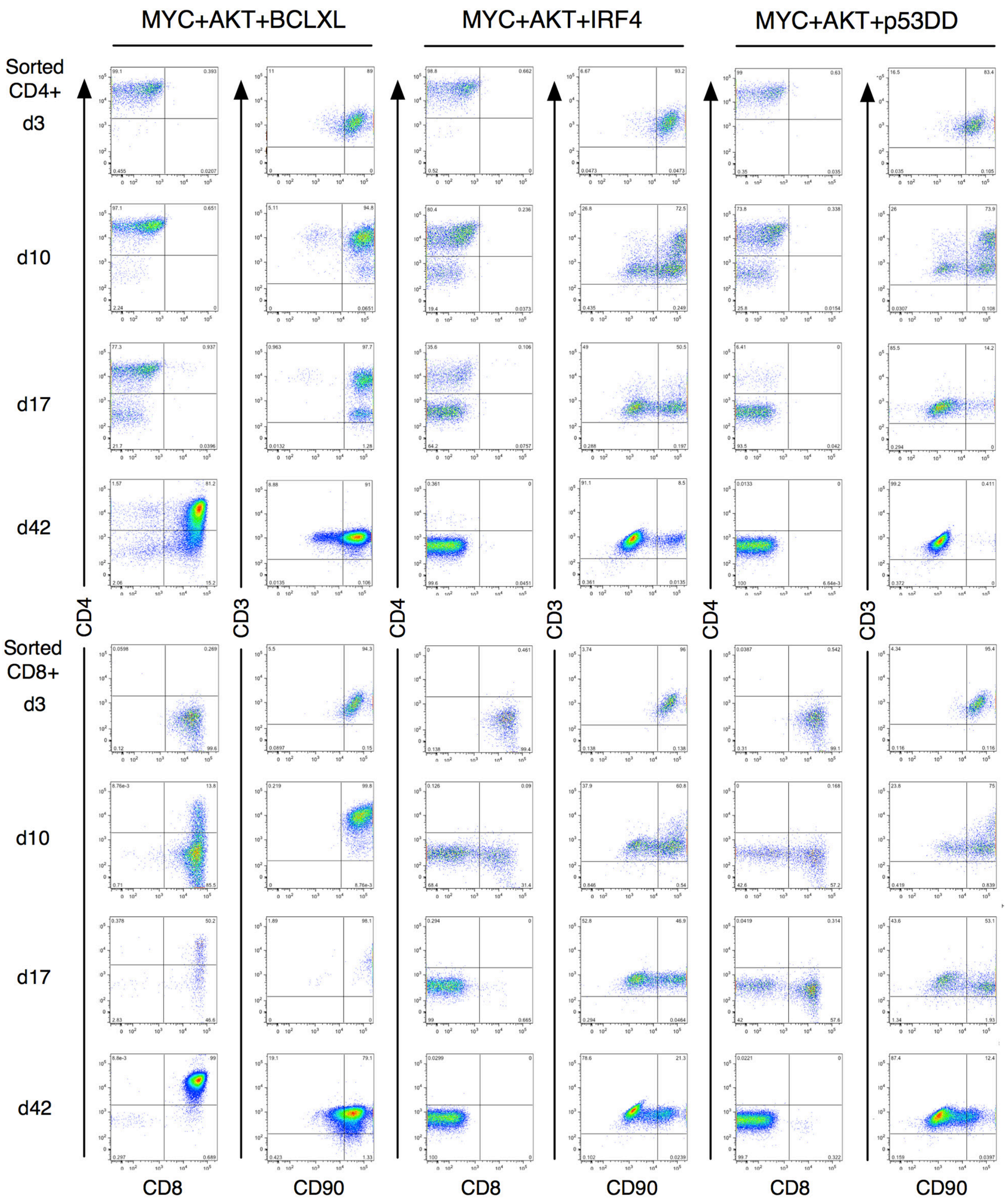

Supplementary Figure 3: Phenotypes of transformed cells sorted for CD4⁺ and CD8⁺ prior to transformation. Spleen cells were sorted into CD4⁺ and CD8⁺ and stimulated with anti-CD3+anti-CD28 for two days followed by transduction with MYC+AKT+BCLXL, MYC+AKT+IRF4, and MYC+AKT+ p53DD encoding retroviruses. Surface expression of CD4, CD8, CD3 and CD90 in transduced cell cultures at indicated days is shown.

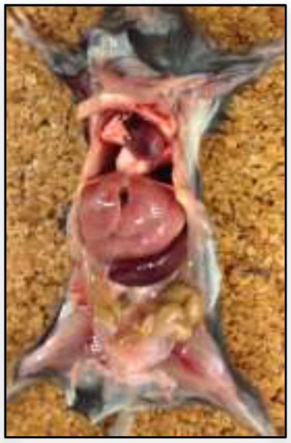

Representative mouse injected with T17 cells

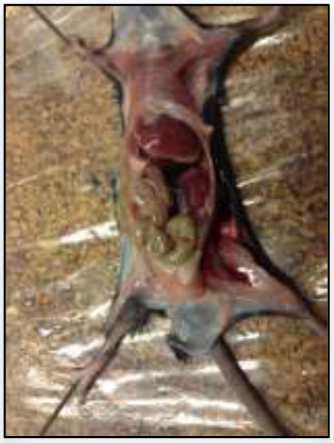

Representative mouse injected with T18 cells

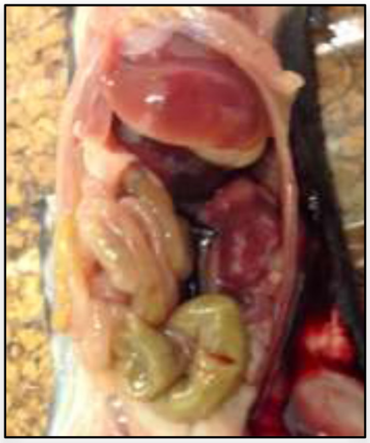

Representative mouse injected with T18 cells (detail)

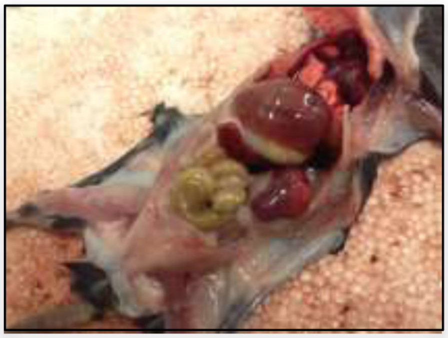

Representative mouse injected with T20 cells

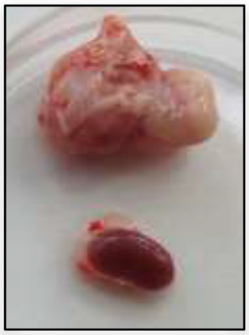

Tumor infiltrated kidney (top) and normal kidney (bottom) from mouse injected with T20 cells

Supplementary Figure 4: Macroscopic aspect of mice injected with transformed T cells. Macroscopic aspect of mice injected with T cells transformed through expression ofMYC+AKT+BCLXL (T17), MYC+AKT+BMI1 (T18) and MYC+AKT+p53DD (T20). Mice injected with T cells transformed with MYC+AKT+IRF4 (T19) did not develop tumors.

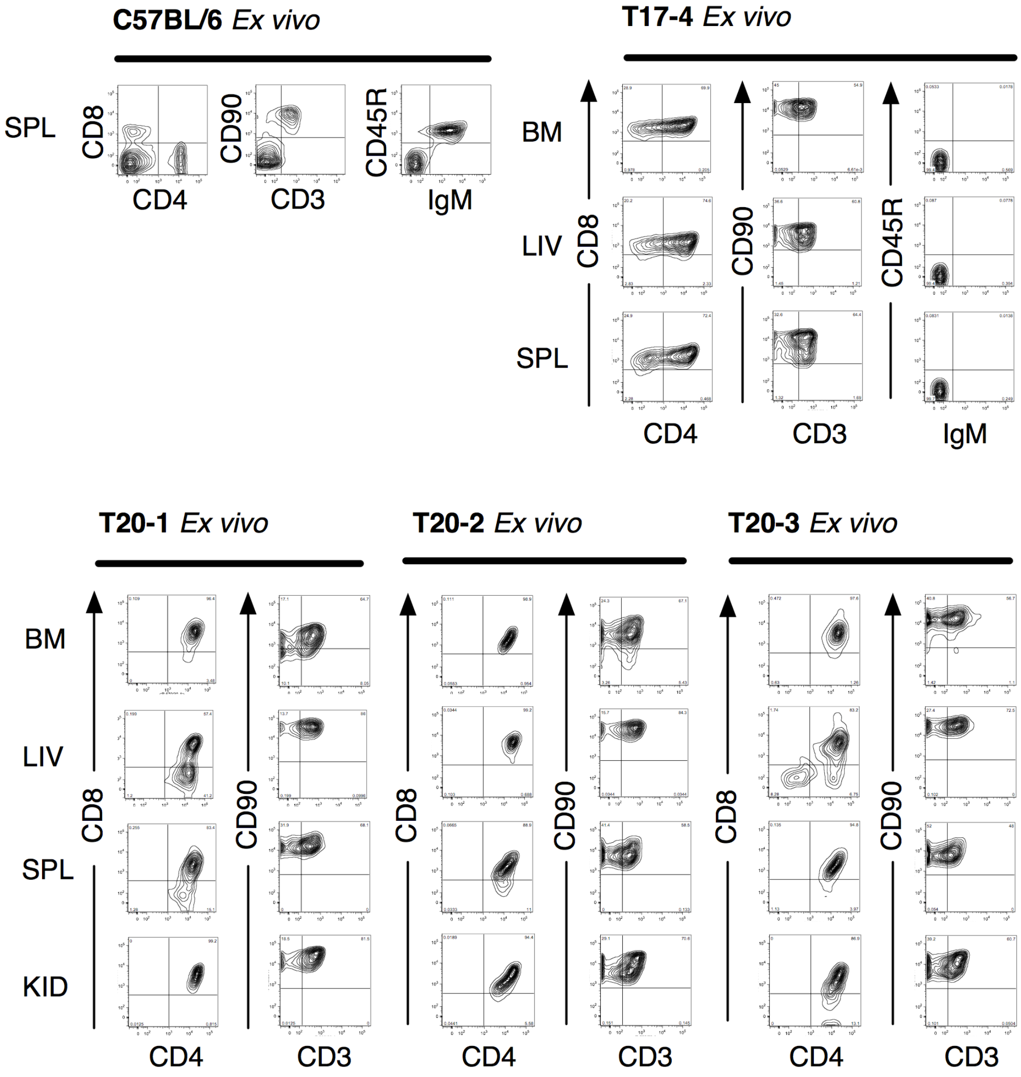

Supplementary Figure 5: Surface phenotypes of tumor cells. Surface phenotypes (CD4, CD8, CD3 and CD90) of infiltrating GFP and YFP positive tumor cells analyzed *ex vivo*. Analysis of spleen cells from C57BL/6 as control and 1 mouse injected with T cells transduced with MYC+AKT+BCLXL and 3 mice injected with T cells transduced with MYC+AKT+p53DD are shown.

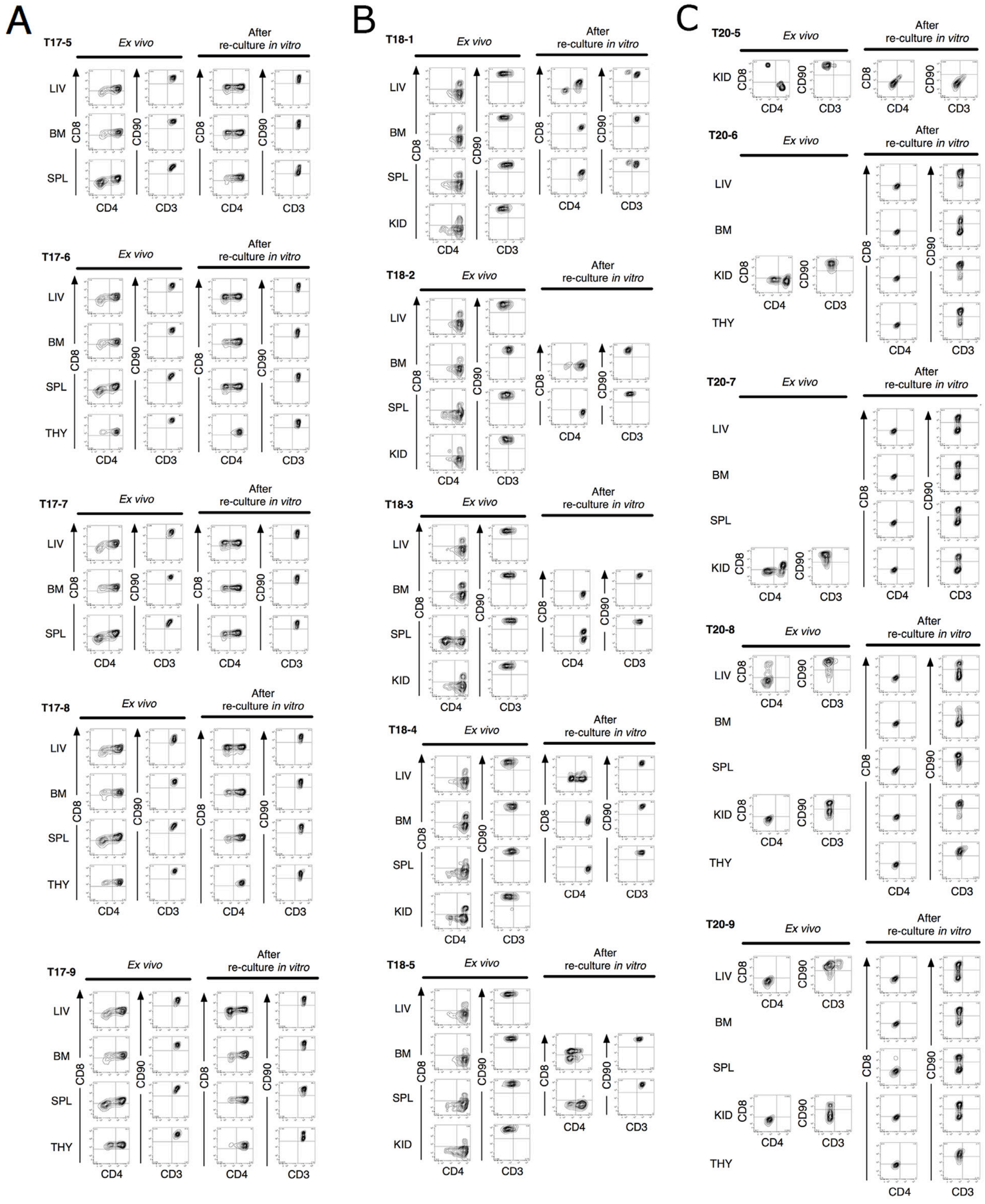

Supplementary Figure 6: (A-C) Surface phenotypes of tumor cells. Surface phenotypes (CD4, CD8, CD3 and CD90) of infiltrating GFP and YFP positive tumor cells analyzed *ex vivo* and after re-culture *in vitro*. Supplementary Figure 6A: Analysis of five mice injected with T cells transduced with MYC+AKT+BCLXL are shown. Supplementary Figure 6B: Five mice injected with T cells transduced with MYC+AKT+BMI1 are shown. Supplementary Figure 6C: Five mice injected with T cells transduced with MYC+AKT+p53DD are shown.

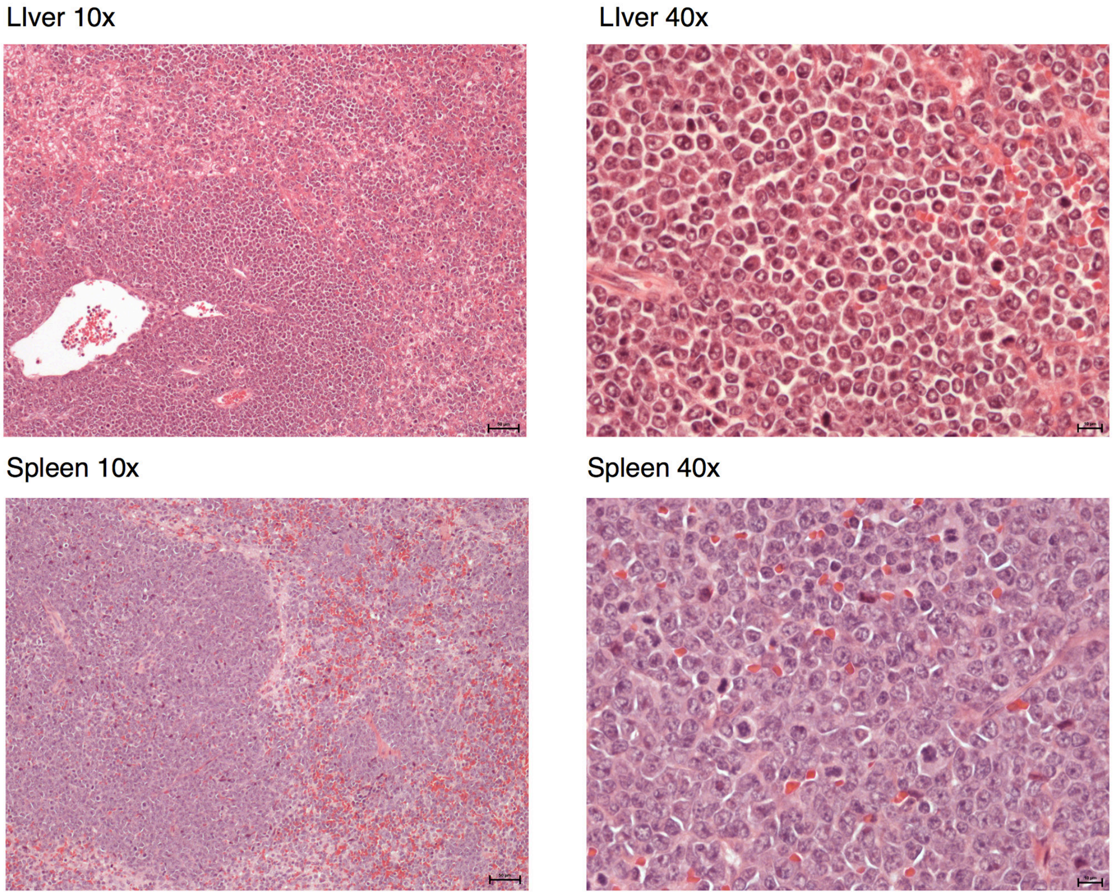

Supplementary Figure 7: Histological analysis of mice injected with transformed T cells (T17). C57BL/6 mice were injected with T cells transformed with MYC+AKT+BCLXL (T17) followed by staining with hematoxylin/eosin of spleen and liver. Histological examination revealed perivascular and sinusoidal lymphomatous infiltration of the liver, expanded white pulp as well as subcapsular lymphocyte invasion in the spleen. Frequent mitoses in many areas were observed. Bars in 10x and 40x indicate 50 µm and 10 µm respectively.

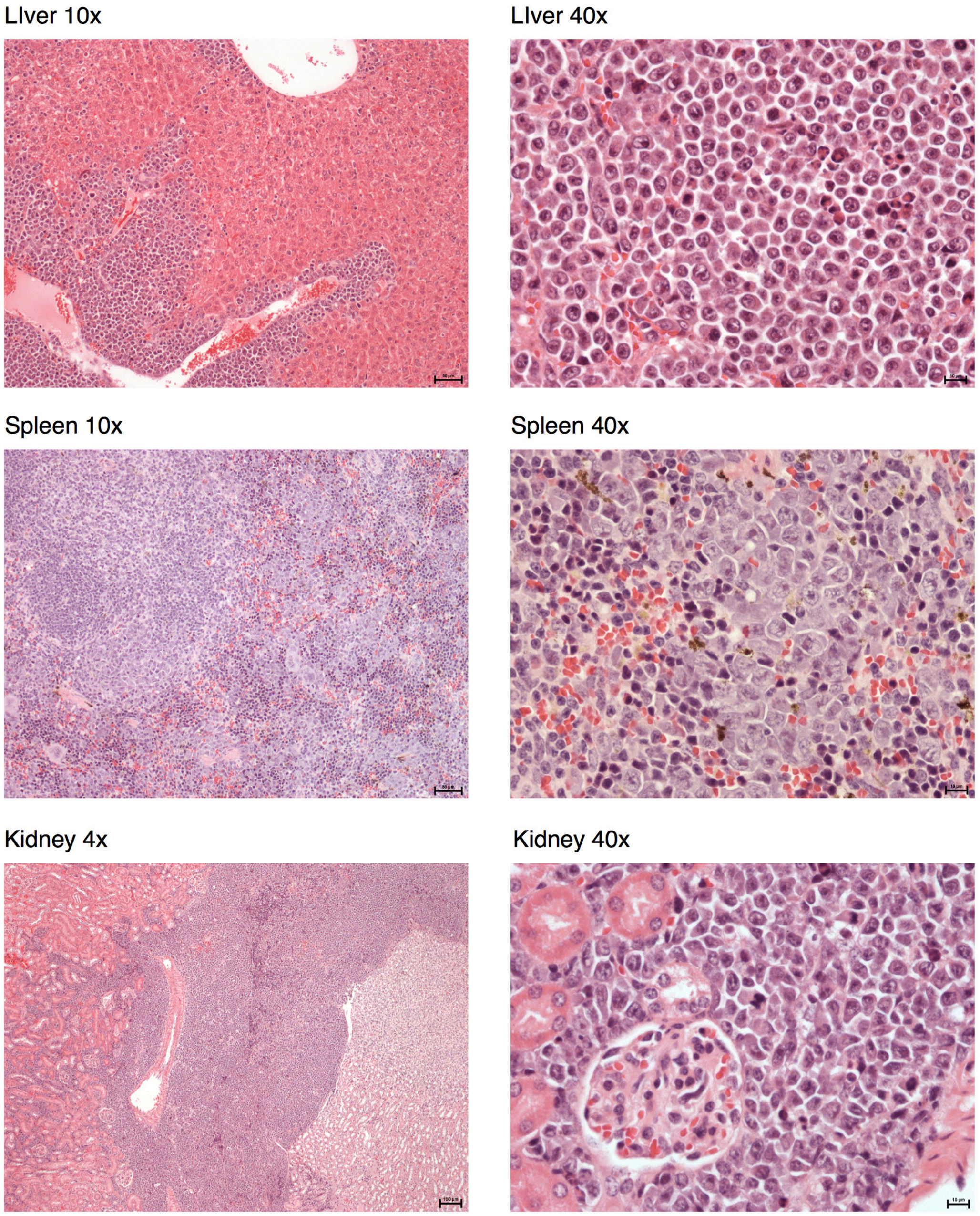

Supplementary Figure 8: Histological analysis of mice injected with transformed T cells (T18). C57BL/6 mice were injected with T cells transformed with MYC+AKT+BMI1 (T18) followed by staining with hematoxylin/eosin of spleen, liver and kidney. Livers displayed marked lymphomatous periportal infiltration occasionally scattered focally in sinusoids. Spleens presented with various degrees of lymphoma invasion in the red and white pulp. Affected kidneys displayed solid lymphoma invasion at the corticomedullary junction, eventually extending between cortical renal tubules and more restrictedly in medullary tubules. There were rather frequent signs of mitoses and apoptotic cells. Bars in 4x, 10x and 40x indicate 100 µm, 50 µm and 10 µm respectively.

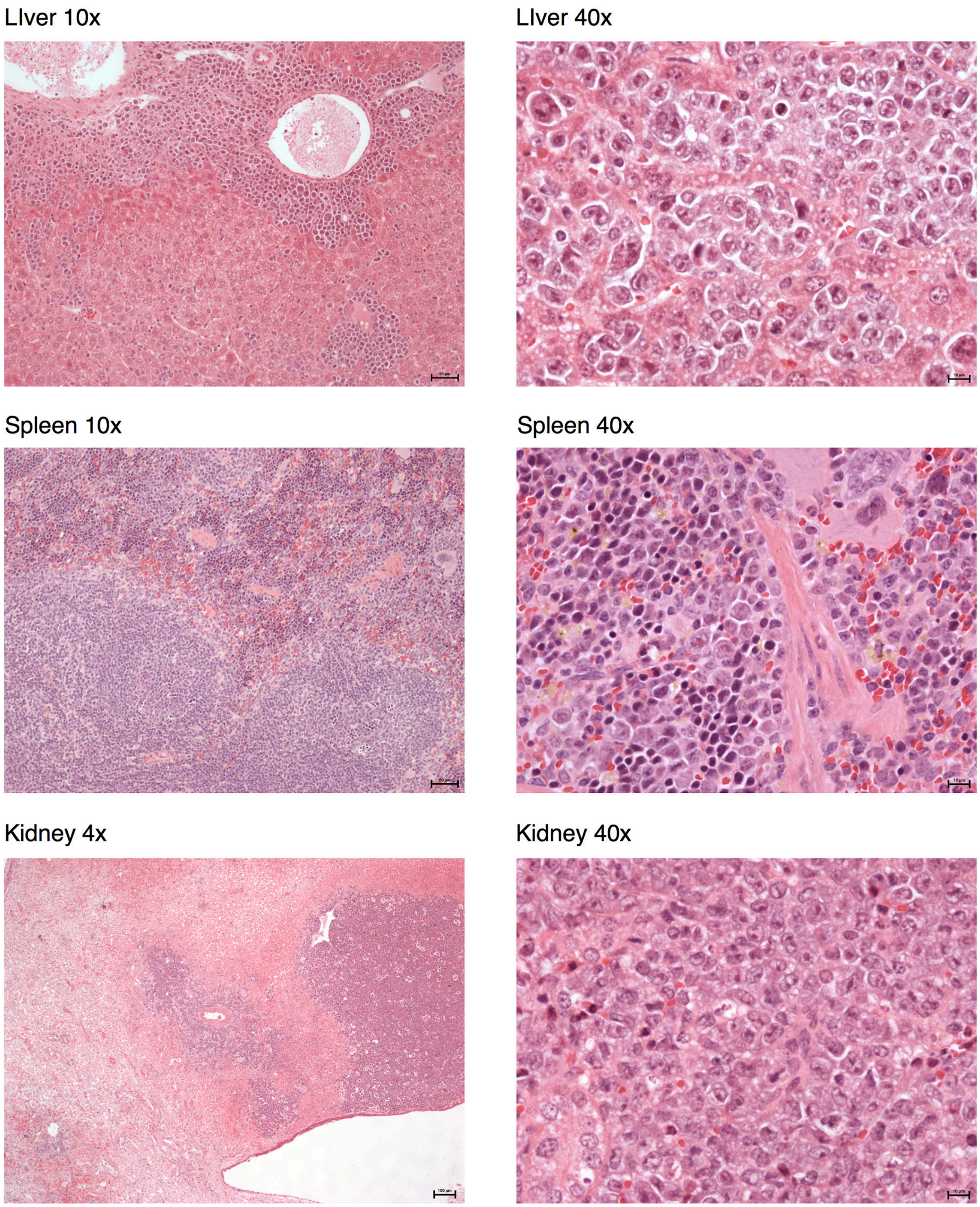

Supplementary Figure 9: Histological analysis of mice injected with transformed T cells (T20). C57BL/6 mice were injected with T cells transformed with MYC+AKT+p53DD (T20) followed by staining with hematoxylin/eosin of spleen, liver and kidney. Histology revealed minor foci of lymphomatous cells in the spleen and liver in two out of five mice. Kidney infiltration ranged from relatively small cortical foci to large lymphomatous infiltrates affecting almost the entire kidney. Bars in 4x, 10x and 40x indicate 100 µm, 50 µm and 10 µm respectively.

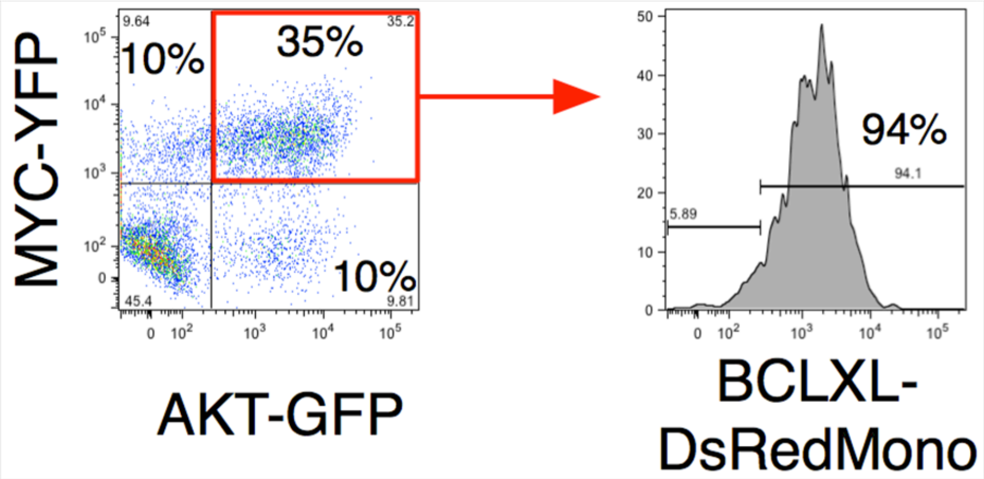

Supplementary Figure 10: Transduction efficiency of T cells prior to injection into mice. Expression of GFP, YFP and DsRed 3 days after transduction, prior to injection into mice.

Supplementary Table 1: Tumor take *in vivo* – T cells transformed with MYC+AKT+BCLXL (T17)

Mouse Id	Organ	Day of death* or sacrifice	% infiltrating cells in liver and kidney**	% GFP/YFP+***	Phenotype GFP/ YFP+ cells analyzed****	Spleen/kidney weight (mg)
T17-1		12*	n.a.	n.a.	n.a.	n.a.
T17-2		12*	n.a.	n.a.	n.a.	n.a.
T17-3		12*	n.a.	n.a.	n.a.	n.a.
T17-4	LIV	14	46.9	96.9	Y	
	KID		n.a.	n.a.	n.a.	
	BM			91.5	Y	
	SPL			29.0	Y	481
T20-1	LIV	28	7.9	59.5	Y	
	KID		37.4	90.4	Y	332/324
	BM			6.6	Y	
	SPL			3.4	Y	190
T20-2	LIV	28	18.3	72.9	Y	
	KID		65.0	94.2	Y	1225/n.a.
	BM			71.6	Y	
	SPL			25.6	Y	308
T20-3	LIV	28	8.3	42.0	Y	
	KID		9.0	86.7	Y	1303/n.a.
	BM			3.7	Y	
	SPL			26.2	Y	243
T20-4	LIV	31	n.a.	n.a.	n.a.	
	KID		n.a.	n.a.	n.a.	287/1270
	BM			n.a.	n.a.	
	SPL			n.a.	n.a.	196

*Found dead in cage.
**As measured by cells in a ”lymphoid” gate based on FCS and SSC.
*** % GFP and YFP expressing cells within the ”lymphoid” gate.
**** Expression of CD4 and CD8 as well as CD3 and CD90 is shown in Supplementary Figure 5. Kidney weights (control): 171/155 mg.

Supplementary Table 2A: Tumor take *in vivo* – T cells transformed with MYC+AKT+BCLXL (T17)

Mouse Id	Organ	Day of sacrifice	% infiltrating cells in liver and kidney*	% GFP/ YFP+**	Phenotype GFP/ YFP+ cells analyzed***	Spleen weight (mg)	Re-growth *in vitro*
T17-5	LIV	9	33.6	98	Y		Y
	KID		n.a.	n.a.	n.a.		n.a.
	BM		-	89	Y		Y
	THY		n.a.	n.a.	n.a.		n.a.
	SPL		-	93.9	Y	272	Y
T17-6	LIV	9	29.1	96.5	Y		Y
	KID		n.a.	n.a.	n.a.		n.a.
	BM		-	87.8	Y		Y
	THY		-	37.5	Y		Y
	SPL		-	89	Y	356	Y
T17-7	LIV	9	27.9	96.5	Y		Y
	KID		n.a.	n.a.	n.a.		n.a.
	BM		-	87.8	Y		Y
	THY		n.a.	n.a.	n.a.		n.a.
	SPL		-	92.1	Y	396	Y
T17-8	LIV	9	29.3	96.7	Y		Y
	KID		n.a.	n.a.	n.a.		n.a.
	BM		-	82.8	Y		Y
	THY		-	53.3	Y		Y
	SPL		-	89.7	Y	431	Y
T17-9	LIV	9	33.2	96.9	Y		Y
	KID		n.a.	n.a.	n.a.		n.a.
	BM		-	82.6	Y		Y
	THY		-	7.8	Y		Y
	SPL		-	89.3	Y	396	Y

*As measured by cells in a ”lymphoid” gate based on FCS and SSC.
**% GFP and YFP expressing cells within the ”lymphoid” gate.
***Expression of CD4 and CD8 as well as CD3 and CD90 is shown in Supplementary Figure 5A.

Supplementary Table 2B: Tumor take *in vivo* – T cells transformed with MYC+AKT+BMI1 (T18)

Mouse Id	Organ	Day of sacrifice	% infiltrating cells*	% GFP/YFP+**	Phenotype GFP/YFP+ cells analyzed***	Spleen weight (mg)	Re-growth *in vitro*
T18-1	LIV	13	9.6	73.8	Y		Y****
	KID		4.9	79.6	Y		N
	BM		-	63.5	Y		Y****
	THY		-	0.2	N		N
	SPL		-	43.7	Y	232	Y****
T18-2	LIV	13	7.7	81.3	Y		N
	KID		1.5	68.4	Y		N
	BM		-	18.1	Y		Y****
	THY		-	0.02	N		N
	SPL		-	12.3	Y	173	Y****
T18-3	LIV	13	11	79.4	Y		N
	KID		5.9	78.8	Y		N
	BM		-	21.8	Y		Y****
	THY		-	0.1	N		N
	SPL		-	15.9	Y	295	Y****
T18-4	LIV	13	8.8	78.6	Y		Y****
	KID		2.2	74.1	Y		N
	BM		-	13.9	Y		Y****
	THY		-	0.01	N		N
	SPL		-	4.5	Y	232	Y****
T18-5	LIV	13	7.2	67.7	Y		N
	KID		3.4	76.8	Y		N
	BM		-	13.2	Y		Y****
	THY		-	0.04	N		N
	SPL		-	2.8	Y	193	Y****

*As measured by cells in a ”lymphoid” gate based on FCS and SSC.
**% GFP and YFP expressing cells within the ”lymphoid” gate.
***Expression of CD4 and CD8 as well as CD3 and CD90 is shown in Supplementary Figure 5B.
****Very difficult to grow *in vitro*. Cells characterized by low viability.

Supplementary Table 2C: Tumor take *in vivo* – T cells transformed with MYC+AKT+p53DD (T20)

Mouse Id	Organ	Day of sacrifice	% infiltrating cells*	% GFP/ YFP+**	Phenotype GFP/YFP+ cells analyzed***	Spleen weight (mg)	Re-growth *in vitro*
T20-5	LIV	20	1.9	0.5	N		N
	KID		6.4	2.2	Y		Y
	BM		-	0.05	N		N
	THY		-	u.d.	N		N
	SPL		-	u.d.	N	71	N
T20-6	LIV	28	3.2	1.6	N		Y
	KID		8.4	60.9	Y		Y
	BM		-	0.04	N		Y
	THY		-	0.02	N		Y
	SPL		-	0.04	N	83	N
T20-7	LIV	28	2.7	1.9	N		Y
	KID		8.8	72.3	Y		Y
	BM		-	0.06	N		Y
	THY		-	u.d.	N		N
	SPL		-	0.11	N	95	Y
T20-8	LIV	34	3.9	36.4	Y		Y
	KID		20.6	81.8	Y		Y
	BM		-	0.7	N		Y
	THY		-	0.01	N		Y
	SPL		-	1.6	N	140	Y
T20-9	LIV	40	6.1	15.9	Y		Y
	KID		6.7	87.8	Y		Y
	BM		-	0.44	N		Y
	THY		-	u.d.	N		Y
	SPL		-	0.26	N	302	Y

*As measured by cells in a ”lymphoid” gate based on FCS and SSC.
**% GFP and YFP expressing cells within the ”lymphoid” gate.
***Expression of CD4 and CD8 as well as CD3 and CD90 is shown in Supplementary Figure 5C.

Supplementary Table 3: Tumor take *in vivo* – T cells transformed with MYC+AKT+BCLXL (T17)

Mouse Id	Organ	Day of sacrifice	% infiltrating cells in liver*	% CD90+**	%EGFP+ EYFP+ DsRed-mono+ among CD90+**	% endo-genous CD90+ EGFP- EYFP- DsRed-mono-**	TcR Vβ Seq.
T17-10	LIV	8	23.8	92.5	93.2	6.8	
	BM		-	42.4	94.4	5.6	
	SPL		-	85.5	92.4	7.6	Y
T17-11	LIV	8	27.2	96.3	93.9	6.1	
	BM		-	57.9	95.0	5	
	SPL		-	93.6	93.3	6.7	Y
T17-12	LIV	8	34.8	96.1	97.4	2.6	
	BM		-	43.1	97.1	2.9	
	SPL		-	87.9	93.6	6.4	
T17-13	LIV	8	34.9	91.8	93.6	6.4	
	BM		-	50.4	96.7	3.3	
	SPL		-	92	90.2	9.8	Y

*% cells in a ”lymphoid” gate based on FCS and SSC.
**% marker expressing cells within the ”lymphoid” gate.

Supplementary Table 4: Summary of TcR-Vβ sequencing

Sample id	Total rearran-gements	Total unique rearran-gements	Total productive rearran-gements	Unique productive rearran-gements	Productive entropy	Productive clonality	Max. productive freq. (%)	Normalized richness
**B6 SPL**	25345	22287	17651	15349	13.8	0.008	0.64	0.870
**Preinj.**	63551	41635	44818	28971	14.5	0.023	0.09	0.646
**T17-10 SPL**	56555	5633	38858	3794	9.4	0.206	4.39	0.098
**T17-11 SPL**	63712	5632	44516	3720	9.5	0.198	3.51	0.084
**T17-13 SPL**	67803	5568	45709	3667	9.8	0.175	1.07	0.080

Productive entropy: Entropy measure for the sample calculated over all Productive Rearrangements. Samples with higher entropy will have a greater diversity of rearrangements while low entropy samples will have many more rearrangements that share nucleotide identity. Productive Entropy is calculated by summing the Productive Frequency times the log (base 2) of the same frequency over all productive rearrangements in a sample.
Productive clonality: Clonality measure for the sample calculated over all Productive Rearrangements. Values for clonality range from 0 to 1. Values near 1 represent samples with one or a few predominant rearrangements (monoclonal or oligoclonal samples) dominating the observed repertoire. Clonality values near 0 represent more polyclonal samples. Productive Clonality is calculated by normalizing Productive Entropy using the total number of unique Productive Rearrangements and subtracting the result from 1.
Max. productive freq: The maximum Productive Frequency value (in %) found within a sample.
Normalized richness: The number of unique productive rearrangements divided by the number of total productive rearrangements.

Supplementary Table 5: TcR-Vβ gene usage

TcR-Vβ gene	B6 SPL	Preinj.	T17-10 SPL	T17-11 SPL	T17-13 SPL
**TCRBV01-01**	5.77	5.40	4.59	6.63	5.28
**TCRBV02-01**	6.02	4.59	2.16	2.40	2.62
**TCRBV03-01**	7.44	8.21	6.52	11.08	6.97
**TCRBV04-01**	4.29	4.55	3.98	3.04	3.98
**TCRBV05-01**	6.12	4.94	6.64	5.67	6.55
**TCRBV10-01**	0.01	0.02	0.01	0.05	0.00
**TCRBV11-01**	0.00	0.00	0.00	0.00	0.00
**TCRBV12-01**	4.25	6.42	6.73	6.48	6.40
**TCRBV12-02**	3.25	4.38	4.23	2.35	3.64
**TCRBV13-01**	5.91	5.49	5.59	4.35	5.16
**TCRBV13-02**	12.22	9.93	10.82	8.47	8.40
**TCRBV13-03**	6.28	6.11	8.19	6.78	10.29
**TCRBV14-01**	2.69	3.21	2.59	2.51	2.36
**TCRBV15-01**	3.55	2.97	2.46	3.33	3.48
**TCRBV16-01**	5.57	6.80	6.19	5.54	10.26
**TCRBV17-01**	2.04	2.70	4.40	6.36	3.67
**TCRBV19-01**	7.80	8.42	13.12	8.47	8.54
**TCRBV20-01**	3.72	3.29	1.95	1.74	2.86
**TCRBV21-01**	0.14	0.17	0.01	0.01	0.00
**TCRBV22-01**	0.15	0.09	0.01	0.01	0.03
**TCRBV23-01**	0.73	0.69	0.95	0.46	0.20
**TCRBV24-01**	1.38	1.27	1.05	0.53	0.97
**TCRBV25-01**	0.00	0.00	0.00	0.00	0.00
**TCRBV26-01**	0.70	0.57	0.27	2.41	1.02
**TCRBV27-01**	0.01	0.00	0.00	0.00	0.00
**TCRBV28-01**	0.00	0.00	0.00	0.00	0.00
**TCRBV29-01**	3.80	5.41	5.70	7.44	5.68
**TCRBV30-01**	0.16	0.19	0.06	0.19	0.11
**TCRBV31-01**	6.00	4.17	1.78	3.70	1.52

Supplementary Dataset 1: Details of TcR-Vβ sequencing results.

See Supplementary File 1
